# Jasmonate‐ and abscisic acid‐activated AaGSW1‐AaTCP15/AaORA transcriptional cascade promotes artemisinin biosynthesis in *Artemisia annua*

**DOI:** 10.1111/pbi.13561

**Published:** 2021-02-14

**Authors:** Ya‐Nan Ma, Dong‐Bei Xu, Xin Yan, Zhang‐Kuanyu Wu, Sadaf Ilyas Kayani, Qian Shen, Xue‐Qing Fu, Li‐Hui Xie, Xiao‐Long Hao, Danial Hassani, Ling Li, Hang Liu, Qi‐Fang Pan, Zong‐You Lv, Pin Liu, Xiao‐Fen Sun, Ke‐Xuan Tang

**Affiliations:** ^1^ Joint International Research Laboratory of Metabolic and Developmental Sciences Key Laboratory of Urban Agriculture (South) Ministry of Agriculture Plant Biotechnology Research Center Fudan‐SJTU‐Nottingham Plant Biotechnology R&D Center School of Agriculture and Biology Shanghai Jiao Tong University Shanghai China; ^2^ Institute of Ecological Agriculture Sichuan Agricultural University Chengdu China; ^3^ Laboratory of Medicinal Plant Biotechnology College of Pharmacy Zhejiang Chinese Medical University Hangzhou China

**Keywords:** *Artemisia annua*, jasmonate, abscisic acid, AaGSW1‐AaTCP15, AaORA transcriptional cascade, artemisinin biosynthesis

## Abstract

Artemisinin, a sesquiterpene lactone widely used in malaria treatment, was discovered in the medicinal plant *Artemisia annua*. The biosynthesis of artemisinin is efficiently regulated by jasmonate (JA) and abscisic acid (ABA) via regulatory factors. However, the mechanisms linking JA and ABA signalling with artemisinin biosynthesis through an associated regulatory network of downstream transcription factors (TFs) remain enigmatic. Here we report AaTCP15, a JA and ABA dual‐responsive teosinte branched1/cycloidea/proliferating (TCP) TF, which is essential for JA and ABA‐induced artemisinin biosynthesis by directly binding to and activating the promoters of *DBR2* and *ALDH1*, two genes encoding enzymes for artemisinin biosynthesis. Furthermore, AaORA, another positive regulator of artemisinin biosynthesis responds to JA and ABA, interacts with and enhances the transactivation activity of AaTCP15 and simultaneously activates *AaTCP15* transcripts. Hence, they form an AaORA‐AaTCP15 module to synergistically activate *DBR2*, a crucial gene for artemisinin biosynthesis. More importantly, *AaTCP15* expression is activated by the multiple reported JA and ABA‐responsive TFs that promote artemisinin biosynthesis. Among them, AaGSW1 acts at the nexus of JA and ABA signalling to activate the artemisinin biosynthetic pathway and directly binds to and activates the *AaTCP15* promoter apart from the *AaORA* promoter, which further facilitates formation of the AaGSW1‐AaTCP15/AaORA regulatory module to integrate JA and ABA‐mediated artemisinin biosynthesis. Our results establish a multilayer regulatory network of the AaGSW1‐AaTCP15/AaORA module to regulate artemisinin biosynthesis through JA and ABA signalling, and provide an interesting avenue for future research exploring the special transcriptional regulation module of TCP genes associated with specialized metabolites in plants.

## Introduction

Malaria, which is caused by *Plasmodium* species (Efferth *et al.,* 2008), threatens humans in many countries and regions of the world every year. There were an estimated 228 million cases and 405 000 related deaths in 2018 (WHO, 2019). Artemisinin (AN), a sesquiterpene lactone, which is extracted from the medicinal plant *Artemisia annua*, is well‐known for its efficient anti‐malaria properties and broad pharmaceutical value. AN‐based combination therapies (ACTs) are the preferred treatment recommended by the World Health Organization (WHO). Chinese scientist Youyou Tu won the Lasker Award in 2011 and the Nobel Prize in 2015 for her discovery of AN in 1972. *A*. *annua* is the only natural source of AN, but its low content (0.01%–1.0% by dry weight) has prompted the semisynthetic biology approach in yeast to produce artemisinic acid (AA), the precursor of AN, using engineered *Saccharomyces cerevisiae* by Keasling’s group (Paddon *et al.,* 2013; Ro *et al.,* 2006). AA is then extracted from fermentation broth and chemically converted to AN. Although the improved engineered yeast was capable of producing 25 g artemisinic acid per litre (Paddon *et al.,* 2013), the yield optimization and commercially relevant concentrations of AA still need to be increased for a viable industrial process, since a high concentration of AA is a prerequisite for the production of high concentrations of AN (Paddon and Keasling, 2014). Moreover, the limited production and high cost of the semisynthetic biology approach in yeast cannot meet worldwide demand and replace the agricultural production of AN at present (Peplow, 2016). Except the semisynthetic biology approach in yeast, a new synthetic biology approach was reported to produce AN using heterologous plant systems. For instance, tobacco plants are applied to produce AN by successfully introducing a core set of genes involved in the mevalonate and the AN biosynthetic pathway separately into the chloroplast and nuclear genomes at the same time (Malhotra *et al.,* 2016), but the AN content 0.8 mg/g dry weight in engineered tobacco is less compared to *A. annua*. Hence, this finding lays a foundation for other alternative host plants except for *A. annua* to produce AN using compartmentalized metabolic engineering.

Substantial evidence suggests that *A*. *annua* possesses two kinds of trichomes including glandular trichomes (GSTs) and T‐shape trichomes (TSTs; Olofsson *et al.,* 2012). Of these, AN is specifically synthesized in the GSTs and is transported to the epicuticular sac at the apex of GSTs (Olofsson *et al.,* 2012; Wang *et al.,* 2016). The AN biosynthetic pathway has almost been elucidated by several groups after years of effort (Figure S1; Bouwmeester *et al.,* 1999; Chang *et al.,* 2000; Paddon *et al.,* 2013; Schramek *et al.,* 2010; Teoh *et al.,* 2009, 2006; Zhang *et al.,* 2008). In summary, the cytosolic mevalonic acid (MVA) pathway and plastidial methylerythritol diphosphate (MEP) pathway‐derived isopentenyl diphosphate (IPP) and isomer dimethylallyl diphosphate (DMAPP) are catalysed by farnesyl diphosphate synthase (FPS) to produce farnesyl diphosphate (FPP), generating the common precursor of terpenoid biosynthesis (Schramek *et al.,* 2010; Towler and Weathers, 2007).

The cyclization of FPP to amorpha‐4, 11‐diene by amorpha‐4, 11‐diene synthase (ADS) is considered as the preliminary step in the AN biosynthetic pathway (Bouwmeester *et al.,* 1999). The next steps are two‐step oxidation of amorpha‐4, 11‐diene to artemisinic alcohol and artemisinic aldehyde by cytochrome P450‐dependent hydroxylase (CYP71AV1) along with NADPH: cytochrome P450 oxidoreductase (CPR) or alcohol dehydrogenase 1 (ADH1; Paddon *et al.,* 2013; Ro *et al.,* 2006; Teoh *et al.,* 2006). The metabolic flux is then divided into two branches from artemisinic aldehyde: one branch involves artemisinic aldehyde being converted to dihydroartemisinic aldehyde via artemisinic aldehyde Δ11(13) reductase (a double‐bond reductase, DBR2) which is a crucial enzyme that efficiently promotes metabolic flux into the AN pathway (Zhang *et al.,* 2008, see Figure S1). Then, dihydroartemisinic aldehyde is catalysed into dihydroartemisinic acid (DHAA) by aldehyde dehydrogenase 1 (ALDH1), and the second branch involves artemisinic aldehyde being converted to AA via ALDH1 (Teoh *et al.,* 2009). Ultimately, DHAA and AA may be transported into the epicuticular sac of GSTs and subsequently converted to AN and arteannuin B (AB) via a light‐induced non‐enzymatic photochemical oxidation process (Brown and Sy, 2004, 2007; Czechowski *et al.,* 2016).

The biosynthesis of specialized metabolites in plants is triggered by several indices such as environmental parameters and exogenous phytohormones. Jasmonic acid (JA) and abscisic acid (ABA) are essential in AN biosynthesis. Nevertheless, the mechanisms of their action are just beginning to be understood. A prior study pointed out that JA enhanced AN yield by activating the pathway structural genes (Maes *et al.,* 2011). Later studies revealed that JA initially induced the expression of some transcription factors (TFs), which positively regulated AN biosynthesis through activating the transcription of AN biosynthetic genes. For instance, AaERF1/2 (ethylene response factor 1/2) and AaTAR1 (trichome and artemisinin regulator 1), which are induced by JA treatment, enhanced the transcripts of AN biosynthetic genes *ADS* and *CYP71AV1* (Tan *et al.,* 2015; Yu *et al.,* 2012). Another JA‐responsive TF AaORA (octadecanoid‐derivative responsive AP2‐domain protein) enhanced the expression of four AN biosynthetic genes *ADS*, *CYP71AV1*, *DBR2* and *ALDH1* (Lu *et al.,* 2013; Ma *et al.,* 2018). In addition, AaWRKY1 and AabHLH1 TFs, which respond to JA treatment, also activated the expression of *ADS* and *CYP71AV1* (Ji *et al.,* 2014; Ma *et al.,* 2009). Notably, elevated AN content by AaMYC2 (myelocytomatosis protein 2), a core activator of JA signalling, had a positive role in JA‐mediation of the specialized metabolites by binding to the *CYP71AV1* and *DBR2* promoters (Shen *et al.,* 2016).

Besides JA, ABA is also reported by several studies to play a vital role in AN production through the activation of structural genes and downstream TFs (Jing *et al.,* 2009; Zhang *et al.,* 2015; Zhong *et al.,* 2018). For example, ABA‐responsive TF AabZIP1 (Basic Leucine Zipper 1) increased *ADS* and *CYP71AV1* expression (Zhang *et al.,* 2015), and ABA‐induced TF AaABF3 activated the *ALDH1* promoter (Zhong *et al.,* 2018). Apart from the individual function of TFs in regulating structural genes, their combinatorial effect to form a transcriptional regulatory cascade is a delicate regulatory strategy at multiple layers. AaGSW1 (glandular trichome‐specific WRKY 1) is a JA and ABA dual‐responsive WRKY TF that activates the promoter regions of *CYP71AV1* and *AaORA*, promoting AN biosynthesis (Chen *et al.,* 2017). Moreover, its transcript is directly regulated by AaMYC2 and AabZIP1, two important regulators of JA and ABA signalling, thus forming AaMYC2/AabZIP1‐AaGSW1‐AaORA transcriptional cascades regulating AN accumulation (Chen *et al.,* 2017), suggesting that the special transcriptional regulatory cascade acts at the nexus of JA and ABA signalling to control AN biosynthesis in *A. annua*. Other transcriptional regulatory modules involved in regulating AN biosynthesis through simultaneously linking JA and ABA signalling need to be identified.

TCP (teosinte branched1/cycloidea/proliferating cell factor) TFs are unique in the plant kingdom. They are divided into two subclasses (class I TCP and class II TCP) according to the TCP domain that is responsible for the specificity of protein‐DNA interactions (Cubas *et al.,* 1999). In *Arabidopsis*, there are 13 class I and 11 class II TCP genes (Cubas *et al.,* 1999). Subsequently, TCP family proteins have been systematically discovered in other plant species, including rice, wheat, tomato, and apple (Parapunova *et al.,* 2014; Xu *et al.,* 2014; Yao *et al.,* 2007; Zhao *et al.,* 2018). Over the past few years, substantial research uncovered diverse mechanisms adopted by TCP proteins to fulfil their broad function. The first mechanisms identified were related to controlling target gene expression by binding to conserved TCP binding sites (TBS; e.g. GGNCCCAC, GCCCR or G(T/C)GGNCCC; Aggarwal *et al.,* 2010). Second, TCP proteins were found to interact with other TCP proteins (Danisman *et al.,* 2012) or with various proteins including TFs (e.g. MYB, ERF, bZIP, NLP), ubiquitin receptors DA1 and DA1‐related proteins (DAR1 and DAR2), and MPK kinase 8 (MPK8; Guan *et al.,* 2017; Nicolas and Cubas, 2016; Peng *et al.,* 2015; Zhang *et al.,* 2019), all of which were partially involved in regulating the transcriptional activities, or protein stability and phosphorylation in a context‐dependent fashion. Additionally, a few studies found that TCP activity was regulated by alternative splicing or microRNA319 (Bresso *et al.,* 2018; Nicolas *et al.,* 2015). However, our knowledge of the transcriptional regulatory factors that control TCP genes at the transcriptional level remains largely unknown.

Correspondingly, functional analyses also indicated that TCP genes have diverse roles in plant growth and development, seed germination, phytohormone signalling, biological clock and plant defence (Doebley *et al.,* 1997; Pruneda‐Paz *et al.,* 2009; Rueda‐Romero *et al.,* 2012; Vadde *et al.,* 2018; Weßling *et al.,* 2014; Zhang *et al.,* 2019, 2017, 2018). Furthermore, the same clade of TCP factors may have functional redundancy (Viola *et al.,* 2016), but the different clades of TCP factors may have an antagonistic effect (Danisman *et al.,* 2012). Moreover, several emerging lines of evidence uncovered the multifaceted role of TCP protein in plant specialized metabolism. In *Arabidopsis*, AtTCP3 promoted flavonoid biosynthesis by enhancing the transactivation activity of R2R3‐MYB proteins, which are components of the R2R3‐MYB/bHLH/WD40 (MBW) ternary complex (Li and Zachgo, 2013). AtTCP15 acted as a repressor in high light‐modulated anthocyanin biosynthesis (Viola *et al.,* 2016). In apple, MdTCP46 promoted high light‐induced anthocyanin accumulation via interaction with MdMYB1 (An *et al.,* 2020). In *Lycium ruthenicum*, LrTCP4 positively regulated kukoamine AN biosynthesis (Chahel *et al.,* 2019). In *A*. *annua*, we previously found that AaTCP14 performed as a positive regulator in AN biosynthesis (Ma *et al.,* 2018). However, the TCP‐related regulatory network associated with plant secondary metabolites has rarely been reported; thus, it is of great interest to further investigate the function of TCP genes in specialized plant metabolism such as biosynthesis of AN.

Here we report that AaTCP15 acts as a positive regulator of JA and ABA‐mediated AN biosynthesis by directly binding to and activating *DBR2* and *ALDH1* promoters. Furthermore, AaORA, a downstream component of JA and ABA signalling involved in promoting AN biosynthesis, exerts a double effect on AaTCP15, including enhancing the transactivation activity of AaTCP15 by direct interaction and elevating expression of *AaTCP15*, leading to the synergistic activation of *DBR2* expression by AaORA‐AaTCP15 module. Strikingly, *AaTCP15* expression is activated by the multiple JA‐ and ABA‐responsive TFs that specifically activate AN biosynthesis. Among them, AaGSW1 acts at the nexus of JA and ABA signalling and directly binds to and activates the *AaTCP15* promoter apart from the reported *AaORA* promoter, and this facilitates formation of the AaGSW1‐AaTCP15/AaORA transcriptional cascade to integrate JA and ABA‐mediated AN biosynthesis. Thus, our data revealed a multilayer transcriptional regulatory cascade of AaGSW1‐AaTCP15/AaORA, which delicately modulates AN biosynthesis through linking the JA and ABA signalling pathways.

## Results

### AaTCP15 acts as a candidate regulator of AN biosynthesis in *A. annua*

Previous studies reported that the plant‐specific TCP TFs normally have similar functions or antagonistic effects (Danisman *et al.,* 2013; Viola *et al.,* 2016). Furthermore, another study showed that *A. annua* AaTCP14 is a positive regulator of AN biosynthesis (Ma *et al.,* 2018), suggesting the possibility of other TCP TFs involved in AN biosynthesis. To identify all putative TCP family genes in *A. annua*, the hidden Markov model (HMM) profiles of the TCP domain (Pfam accession No.: PF03634) were used as queries against the local GST transcriptome database and annotated genome database (Shen *et al.,* 2018) in the HMMER3.0 search program (Finn *et al.,* 2011). Next, the obtained candidate sequences were used as queries against the conserved domain database (https://www.ncbi.nlm.nih.gov/Structure/cdd/wrpsb.cgi). After removing redundant sequences and sequences without the typical TCP domain, we obtained 38 full‐length TCP genes in *A. annua*.

**Figure 1:**
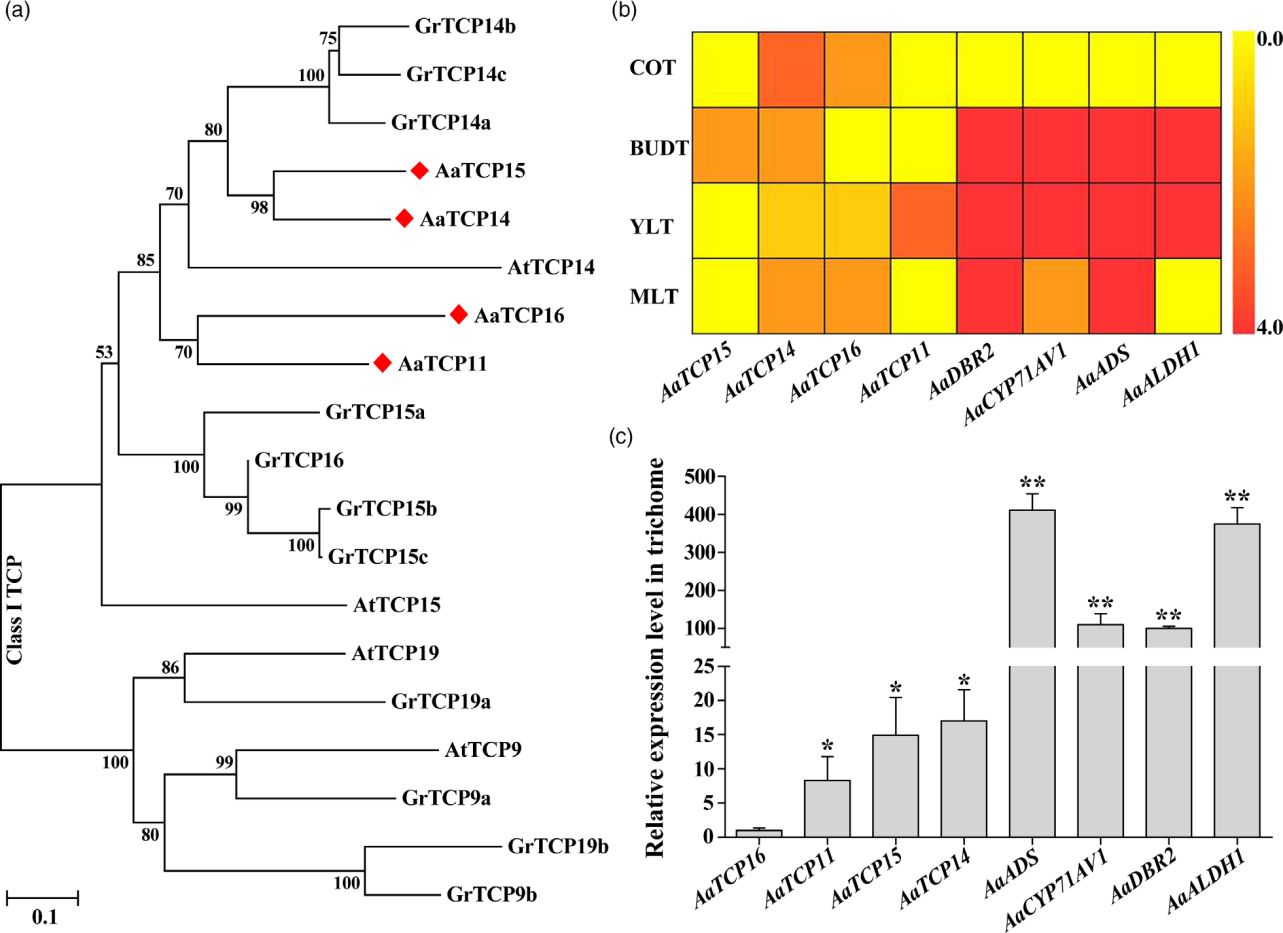
Identification of AaTCP15. (a) Phylogenetic tree showing the relationships of class I TCP transcription factors in *Artemisia annua*, *Arabidopsis thaliana* and *Gossypium raimondii*. TCP sequences were aligned with Clustal W, and a neighbour‐joining phylogenetic tree was constructed using MEGA. Bootstrap values are based on 2000 replicates. AaTCP14, AaTCP11, AaTCP15 and AaTCP16 are marked with red diamonds. The amino acid sequences are named based on the species name. (b) The heat map of transcriptome data includes indicated genes with differential expression in cotyledon (COT), flower bud trichomes (BUDT), young leaf trichomes (YLT) and mature leaf trichomes (MLT) of *Artemisia annua*. The colour scale at the right represents the value of transformed reads per kilobase per million mapped reads. (c) Relative expression levels of *AaTCP16*, *AaTCP11*, *AaTCP15*, *AaTCP14*, *AaADS*, *AaCYP71AV1*, *AaDBR2* and *AaALDH1* in trichomes were measured by quantitative real‐time PCR (qRT‐PCR). The expression level of *AaTCP16* was set as 1. *AaActin* was used as an internal control. The data represent the means ± SD of three replicates from three independent *A. annua* plants. *P < 0.05, **P < 0.01, Student’s *t*‐test.

To explore the evolutionary and phylogenetic relationships between *A. annua* TCP genes and other known TCPs, and also to characterize how the potential *A. annua* TCP genes functioned in AN biosynthesis, an unrooted neighbour‐joining (NJ) tree was constructed using 38 TCP genes from *A. annua*, 24 TCP genes from *Arabidopsis*, and 38 TCP genes from *Gossypium raimondii* (Figure S2). Based on the bootstrap values of clades and the topology of the tree, three candidate class I TCP proteins, AaTCP11, AaTCP15 and AaTCP16, were distributed into the same clade with AaTCP14, a pivotal regulator of AN biosynthesis (Figures 1a and S2). Among these, AaTCP15 clustered closest with AaTCP14 and contained a generally conserved TCP domain (Figure S3). Next, to screen one out of the three TCP genes that is mostly associated with AN biosynthesis, the heatmaps of expression levels of *AaTCP11*, *AaTCP15*, *AaTCP16*, *AaTCP14* and AN biosynthetic genes *ADS*, *CYP71AV1*, *DBR2* and *ALDH1* were preliminarily analysed based on the early published transcriptome database from different *A. annua* tissues (Graham *et al.,* 2010). Results revealed partial similarity with *ADS*, *CYP71AV1*, *DBR2* and *ALDH1*, and expression of *AaTCP15*, *AaTCP16* and *AaTCP11* was also detected in trichomes (Figure 1b), where AN is mainly synthesized and accumulated in *A. annua*. In addition, quantitative RT‐PCR (qRT‐PCR) in the trichomes of *A. annua* was carried out to re‐evaluate the expression levels of *AaTCP11*, *AaTCP15*, *AaTCP16*, *ADS*, *CYP71AV1*, *DBR2* and *ALDH1*. Results showed that, similar with the above heatmap result, all of them were expressed in trichomes, and the expression level of *AaTCP15* was higher in trichomes compared with *AaTCP11* and *AaTCP16* (Figure 1c). Thus, according to the phylogenetic relationships and the results of the expression profiles of candidate TCP genes, AaTCP15 was chosen for further research due to its potential role in AN biosynthesis in *A. annua*.

### Expression profiles and nuclear localization of AaTCP15

**Figure 2:**
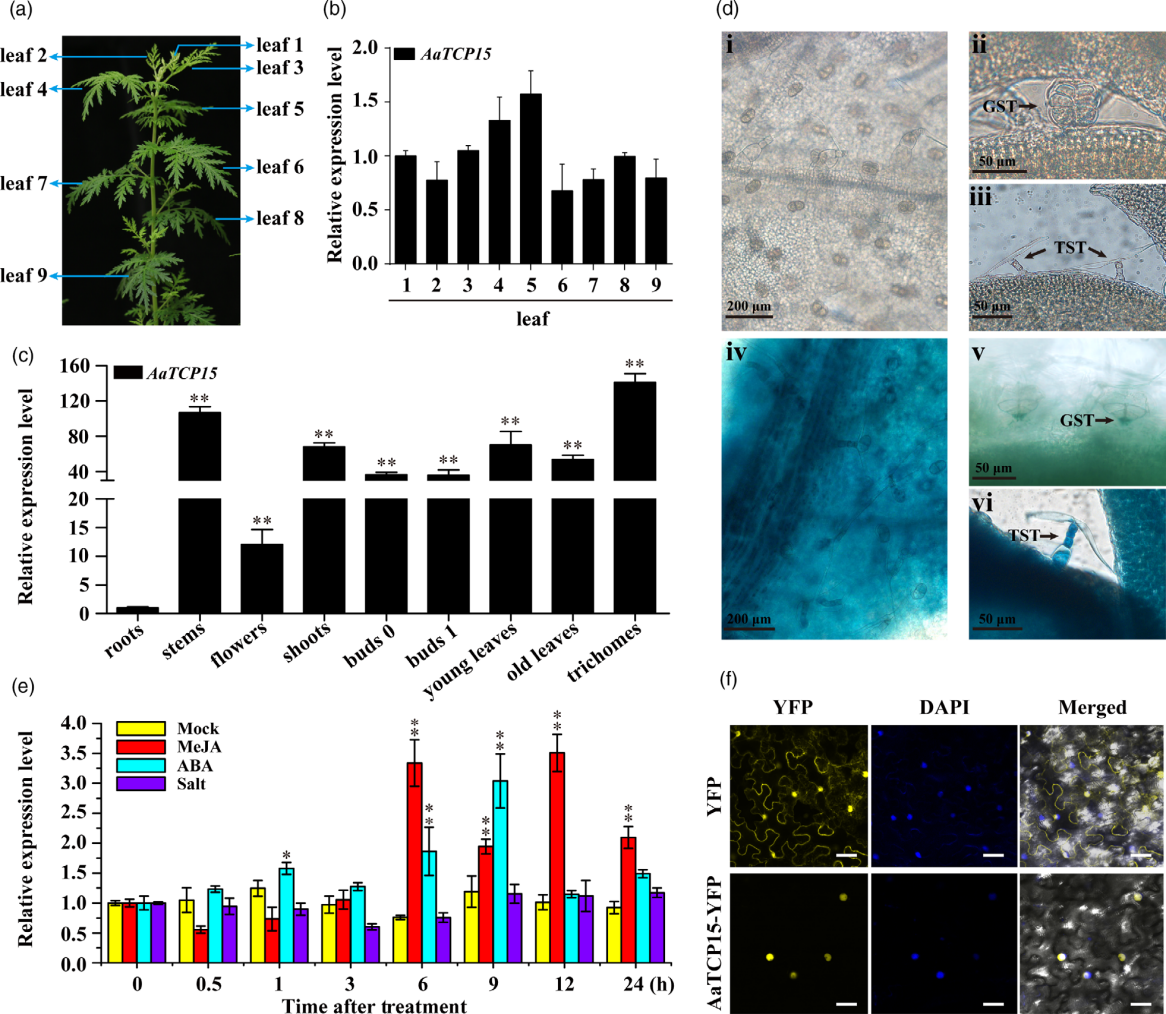
Expression pattern and subcellular localization of AaTCP15. (a) A schematic diagram of the labelled *A. annua* leaves used in quantitative real‐time PCR (qRT‐PCR) assays in (b). (b) Relative expression levels of *AaTCP15* in leaves at different positions. The expression level of *AaTCP15* in leaf 1 was set as 1. *AaActin* was used as an internal control. The data represent the means ± SD of three replicates from three independent *A. annua* plants. (c) Relative expression levels of *AaTCP15* in roots, stems, flowers, shoots, buds (buds 0 and 1), leaves (young leaves and old leaves) and trichomes were measured by qRT‐PCR. The expression level of *AaTCP15* in roots was set as 1. *AaActin* was used as an internal control. The data represent the means ± SD of three replicates from three independent *A. annua* plants. **P < 0.01, Student’s *t*‐test. (d) *GUS* expression (blue staining) in *A. annua* plants transformed with the *1391Z‐GUS* empty vector (control plants) and *1391Z*‐*proTCP15*‐*GUS*. (i, ii and iii), leaves of control plants. (iv, v and vi), leaves of *1391Z*‐*proTCP15*‐*GUS* transgenic plants. TST, T‐shape trichome; GST, glandular trichome. Bars represent 200 μm in (i, iv) and 50 μm in (ii, iii, v, vi). (e) Relative expression levels of *AaTCP15* in the leaves of *A. annua* plants treated with mock (0.1% ethanol), MeJA (100 μm), ABA (100 μm) and NaCl (150 mm) at the indicated times. *AaActin* was used as an internal control. The data represent the means ± SD of three replicates from three independent experiments. *P < 0.05, **P < 0.01, Student’s *t*‐test. (f) Subcellular localization of AaTCP15 in tobacco leaf epidermal cells. The nucleus was determined by 4′, 6‐diamidino‐2‐phenylindole (DAPI) staining. Yellow fluorescent protein (YFP) was used as a negative control. Three independent transfection experiments were performed. Scale bar = 20 μm.

To further investigate *AaTCP15* expression, we examined the expression pattern of *AaTCP15* in leaves at different positions and in different tissues by qRT‐PCR. The analysis showed that *AaTCP15* was broadly expressed in different leaves, with the highest expression in leaf 5 followed by leaf 4, and relatively lower expression in leaf 2, leaf 6, leaf 7 and leaf 9 compared to leaf 1, whereas the expression level of *AaTCP15* in leaf 3 and leaf 8 is comparable to leaf 1 (Figure 2a,b). Furthermore, the *AaTCP15* transcript was also detected in different *A. annua* tissues, with the highest expression in trichomes (Figure 2c). To analyse the sites of *AaTCP15* expression, we generated *AaTCP15* promoter‐*GUS* fusion (*1391Z*‐*ProTCP15*‐*GUS*) transgenic *A. annua* plants and tested their GUS activity. Results revealed that GUS activity was observed in mesophyll cells in young leaves, and *GUS* expression was also observed in the two types of trichomes (TSTs and the two basal cells of GSTs; Figure 2d, iv, v and vi). No GUS activity was detected in *A. annua* plants transformed with the *1391Z‐GUS* empty vector (control plants; Figure 2d, i, ii and iii). These results are in accordance with the previous expression pattern in trichomes (Figures 1c and 2c), indicating that AaTCP15 may function in the trichomes where the specialized metabolites are synthesized and stored.

Numerous reports have shown that methyl jasmonate (MeJA), ABA and salt stress are crucial mediators in AN biosynthesis by activating downstream regulatory genes (Jing *et al.,* 2009; Li *et al.,* 2014; Maes *et al.,* 2011; Paul and Shakya, 2013; Zhou and Memelink, 2016). Hence, we examined whether *AaTCP15* was regulated by these treatments. Based on qRT‐PCR analysis, the *AaTCP15* transcript was induced by MeJA and ABA, respectively (Figure 2e). It is notable that the *AaTCP15* transcript level displayed a delayed increment after prolonged MeJA exposure (6, 9, 12, and 24 h), that peaked at 12 h. In addition, *AaTCP15* expression was increased at 1 h after ABA exposure and significantly increased and reached its highest level after 9 h, then returned to the original levels with prolonged ABA exposure (12 and 24 h). By contrast, the *AaTCP15* transcript showed no differences under either mock or salt treatment (Figure 2e). These results suggested that *AaTCP15* responds to JA and ABA signalling, which may be a potential downstream component of JA and ABA signalling in regulation of AN biosynthesis.

To determine the subcellular localization of AaTCP15, we transiently expressed an AaTCP15‐YFP (yellow fluorescent protein) fusion protein under the control of cauliflower mosaic virus (CaMV) 35S promoter in *Nicotiana benthamiana* leaf cells. As shown in Figure 2f, the recombinant AaTCP15‐YFP fusion protein was specifically localized to the nucleus in *N*. *benthamiana* leaf cells. This result showed that AaTCP15 is a nuclear‐localized protein, consistent with the role of AaTCP15 as a TF.

### AaTCP15 enhances AN biosynthesis and is essential for JA‐ and ABA‐mediated AN biosynthesis in *A. annua*

**Figure 3:**
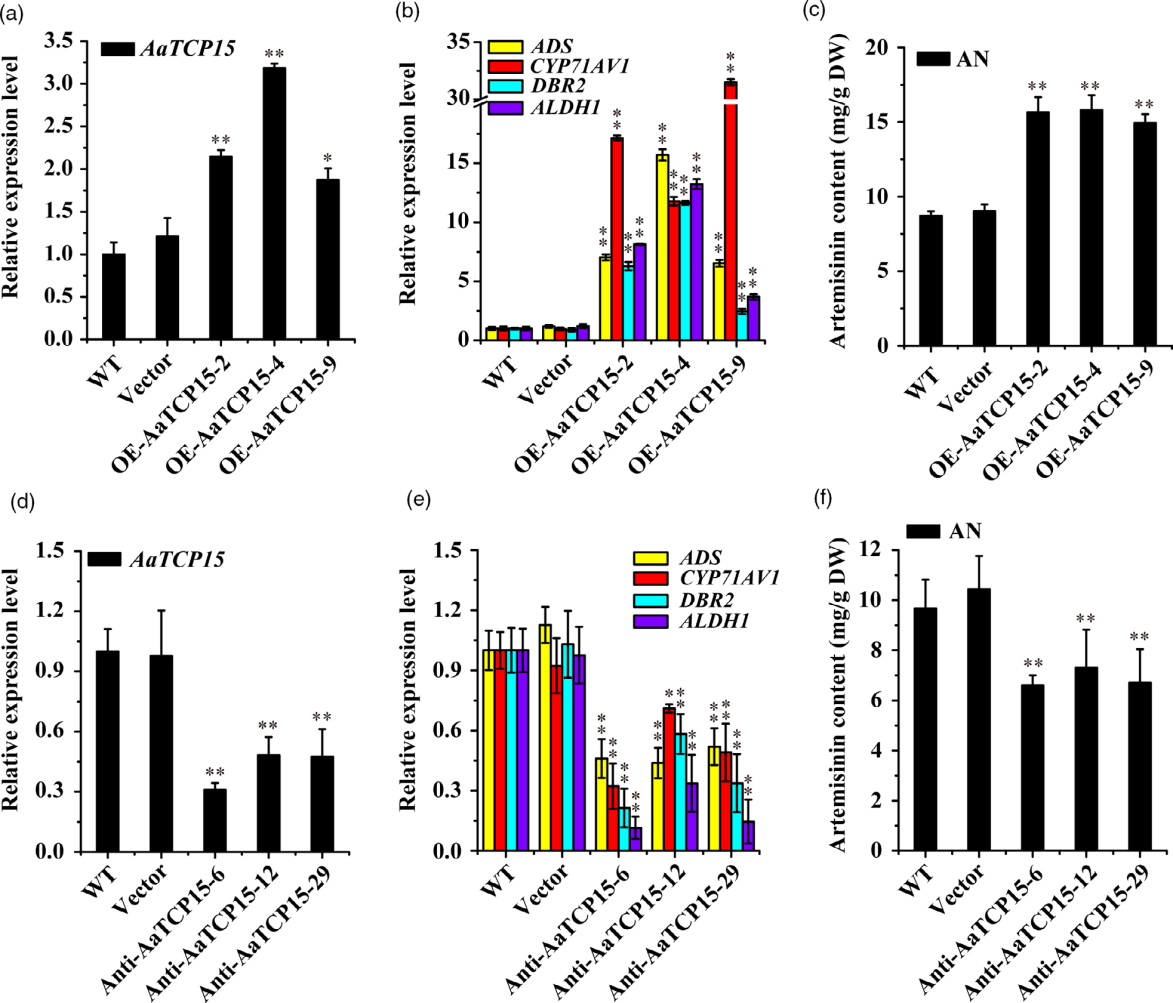
*AaTCP15* transgenic plants modulate artemisinin biosynthesis. (a, d) Expression levels of *AaTCP15* in the leaves of different *A. annua AaTCP15*‐overexpression (OE‐AaTCP15) (a) and *AaTCP15*‐antisense (Anti‐AaTCP15) lines (d), plants transformed with the empty vector (control plants, labelled as Vector) and wild‐type (WT) plants. *AaActin* was used as the internal standard. (b, e) Expression levels of *ADS*, *CYP71AV1*, *DBR2* and *ALDH1* in the leaves of different *A. annua* OE‐AaTCP15 (b) and Anti‐AaTCP15 lines (e), Vector control and WT plants. *AaActin* was used as the internal control. (c, f) HPLC analysis of artemisinin (AN) content in the leaves of different *A. annua* OE‐AaTCP15 (c) and Anti‐AaTCP15 lines (f), Vector control and WT plants. All data represent the means ± SD of three replicates from three cutting propagations. *P < 0.05, **P < 0.01, Student’s *t*‐test.

To assess the biological role of AaTCP15 in controlling AN biosynthesis, stable *AaTCP15*‐overexpression (OE‐AaTCP15) and antisense (Anti‐AaTCP15) transgenic *A. annua* lines were generated. After testing the levels of *AaTCP15* mRNA in these transgenic lines, we selected three independent overexpression lines (designated as OE‐AaTCP15‐2, 4, 9) or three antisense lines (designated as Anti‐AaTCP15‐6, 12, 29) for further characterization (Figure 3a,d). Results showed that, compared to the wild‐type (WT) and Vector controls (*A. annua* plants transformed with the empty vector), the expression levels of AN biosynthetic genes (*ADS*, *CYP71AV1*, *DBR2* and *ALDH1*) and AN content followed the change of *AaTCP15* expression, in that they were significantly up‐regulated in OE‐AaTCP15 (Figure 3a–c) and down‐regulated in Anti‐AaTCP15 *A. annua* lines (Figure 3d–f), suggesting a positive role of AaTCP15 in AN biosynthesis. However, we found that the dihydroartemisinic acid (DHAA) content was decreased in OE‐AaTCP15 and increased in anti‐AaTCP15 transgenic *A. annua* lines, compared to Vector controls (Figure S4a,c). This phenomenon may be attributed to the role of AaTCP15 in regulating some potential as‐yet unknown proteins involved in affecting the photo‐oxidation process from DHAA to AN. Moreover, no obvious morphological differences were observed in either the OE‐AaTCP15 or anti‐AaTCP15 transgenic plants compared with Vector controls (Figure S4b,d). Collectively, these results indicated that AaTCP15 enhanced AN biosynthesis through activating the AN biosynthetic genes.

Subsequently, the function of AaTCP15 in JA‐ and ABA‐mediated AN biosynthesis was investigated. As expected, application of exogenous MeJA and ABA treatments increased AN accumulation significantly in the WT and Vector samples compared to Mock treatment (Figure S5), in accordance with a prior report (Maes *et al.,* 2011). In addition, suppression of *AaTCP15* significantly inhibited JA or ABA‐induced AN accumulation compared to the WT and Vector controls (Figure S5), further supporting that AaTCP15 was essential for JA‐ and ABA‐mediated AN accumulation in *A. annua*.

### AaTCP15 enhances the transcription of both *DBR2* and *ALDH1* by binding to their promoters

**Figure 4:**
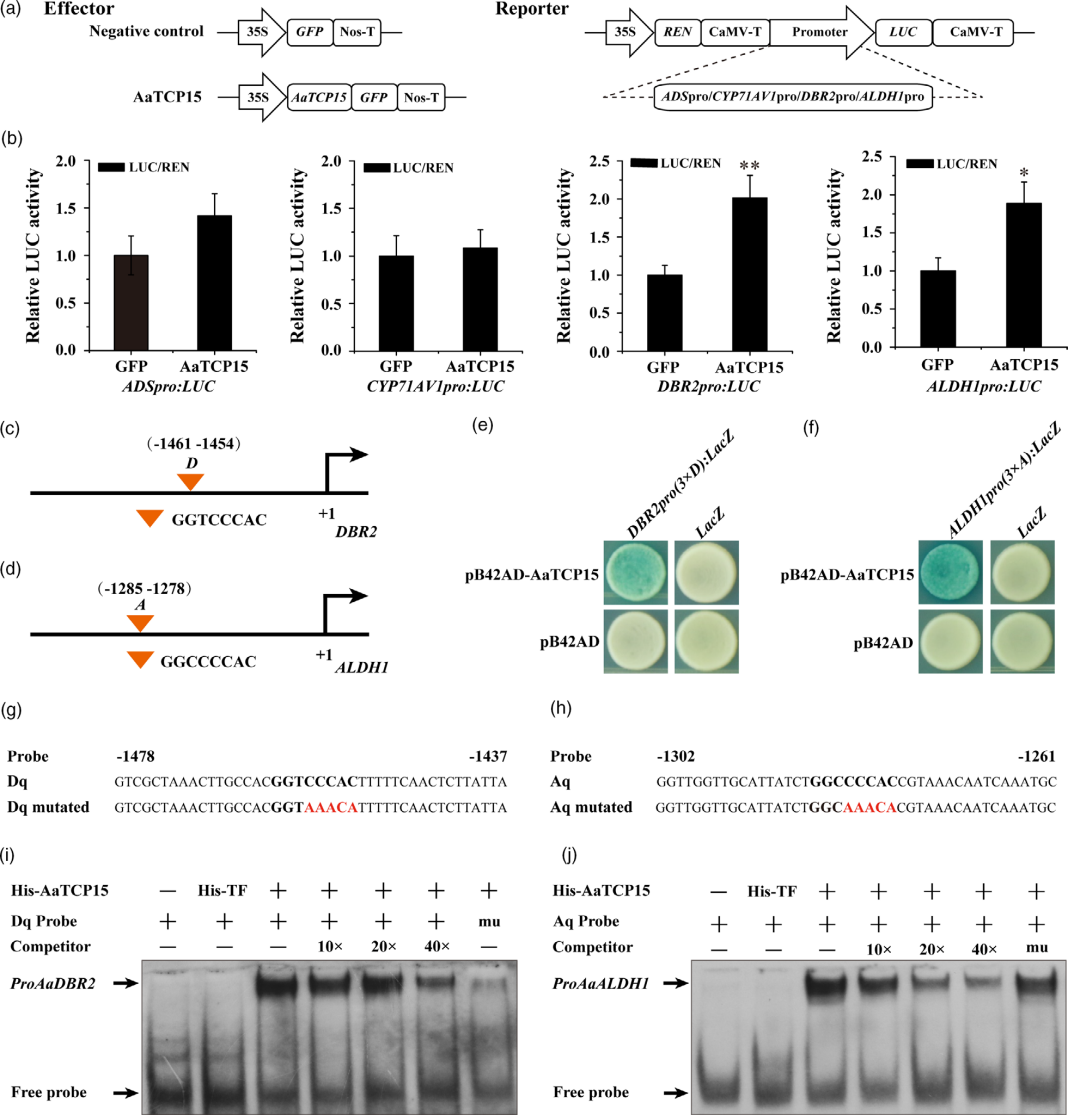
AaTCP15 is a transcriptional activator of *DBR2* and *ALDH1*. (a) Schematic diagrams of the effector and reporter plasmids used in Dual‐LUC assays. REN, *Renilla* luciferase. LUC, firefly luciferase. (b) Dual‐LUC assay in *N. benthamiana* leaf cells using the constructs shown in (a). The GFP effector was used as a negative control, and the LUC/REN ratios of GFP were set as 1. Three independent transfection experiments were performed. The data represent the means ± SD of three replicates from three independent experiments. *P < 0.05, **P < 0.01, Student’s *t*‐test. (c, d) Schematic diagrams of the *DBR2* (c) and *ALDH1* (d) promoters. The positions of potential TBS (TCP binding site) DNA binding sites (*D* in *DBR2* promoter and *A* in *ALDH1* promoter) are shown as orange triangles and are numbered based on their distance from the translational start site (ATG), which is set as +1. (e, f) Y1H assays showing that AaTCP15 binds to the TBS motifs of *DBR2* and *ALDH1*. Three tandem repeats of *D* or *A* motifs were used as baits. Transformed yeast cells were grown on selective medium SD/‐Trp/‐Ura containing 20 mg/L X‐gal, and pictures were taken after 4 days of incubation at 30 °C. Blue plaques indicate protein‐DNA interactions. The Y1H assays were repeated three times, and representative results are shown. (g, h) The sequences of wild‐type (Dq or Aq) and mutated probes (Dq mutated or Aq mutated) used for EMSAs corresponding to *DBR2* or *ALDH1* promoter. Class I TCPs binding motifs are shown in bold, and the mutated nucleotides are indicated by red. (i, j) EMSA assays showing that AaTCP15 binds to the *Dq* motif from *DBR2* and the *Aq* motif from *ALDH1*. Unlabelled Dq, Aq and mutated Aq probes were used as cold competitors, and labelled mutated Dq probe was tested as a control. 10×, 20×, 40× indicate the fold excess of cold competitor relative to that of labelled probe. His‐TF protein was used as a negative control.

To unravel the mechanism of how AaTCP15 regulated the expression of AN biosynthetic genes, we first carried out transient dual‐luciferase (Dual‐LUC) assays. The reporters (*ADSpro*/*CYP71AV1pro*/*DBR2pro*/*ALDH1pro*:*LUC*) along with the effectors (*35S:GFP* or *35S:AaTCP15*, see Figure 4a) were transiently co‐expressed in the *N. benthamiana* leaf cells. As shown in Figure 4b, AaTCP15 only significantly activated the *DBR2* or *ALDH1* promoter compared to the GFP control (Figure 4b).

In plants, TCP TFs regulate their target genes by binding to the conserved promoter motifs, namely TBSs (Aggarwal *et al.,* 2010). Our previous research uncovered that AaTCP14 could bind to *DBR2* and *ALDH1* promoters and activate their expression (Ma *et al.,* 2018), and bioinformatics analysis revealed the presence of TBSs including *D* motif or *A* motif in the *DBR2* or *ALDH1* promoter (Figure 4c,d). Thus, we speculated that AaTCP15, which is closely homologous with AaTCP14 based on a phylogenetic analysis (Figure 1a), may also directly bind to the *DBR2* or *ALDH1* promoter. Yeast one‐hybrid assay showed that binding of the pB42AD‐AaTCP15 fusion protein, rather than pB42AD alone, to three tandem repeats of the *D* motif from *DBR2* promoter or *A* motif from *ALDH1* promoter, strongly activated the expression of the *LacZ* reporter gene (Figure 4e,f), indicating that AaTCP15 binds to the *D* or *A* motifs in the *DBR2* or *ALDH1* promoters, respectively.

Next, electrophoretic mobility shift assays (EMSAs) were conducted to verify AaTCP15 binding to *DBR2* and *ALDH1* promoters using His‐AaTCP15 fusion protein or His‐TF (trigger factor) and *DBR2* or *ALDH1* promoter probe (normal Dq or Aq probe, and mutated Dq or Aq probe, see Figure 4g,h). As shown in Figure 4i,j, a single shift band was detected in the presence of both His‐AaTCP15 and labelled Dq or Aq probe containing the *D* or *A* motif (Figure 4g,h), rather than the combination of His‐TF and labelled Dq or Aq probe; the intensity of this band decreased with increasing concentrations of cold competitor (unlabelled Dq or Aq probe; Figure 4i,j). Moreover, the His‐AaTCP15 protein was unable to bind to the labelled mutated Dq probe referred to as the *DBR2* promoter (Figure 4i). Similarly, using the excess unlabelled mutated Aq probe as a cold competitor could not affect the binding of His‐AaTCP15 to the labelled Aq probe in the *ALDH1* promoter (Figure 4j). Together, these results strongly supported the notion that AaTCP15 activated *DBR2* and *ALDH1* expression via specifically binding to the TBSs element (*D* or *A* motif) in their promoters.

### **AaTCP15 directly interacts with AaORA and synergistically activates *DBR2* transcription by protein**‐**protein interaction**

**Figure 5:**
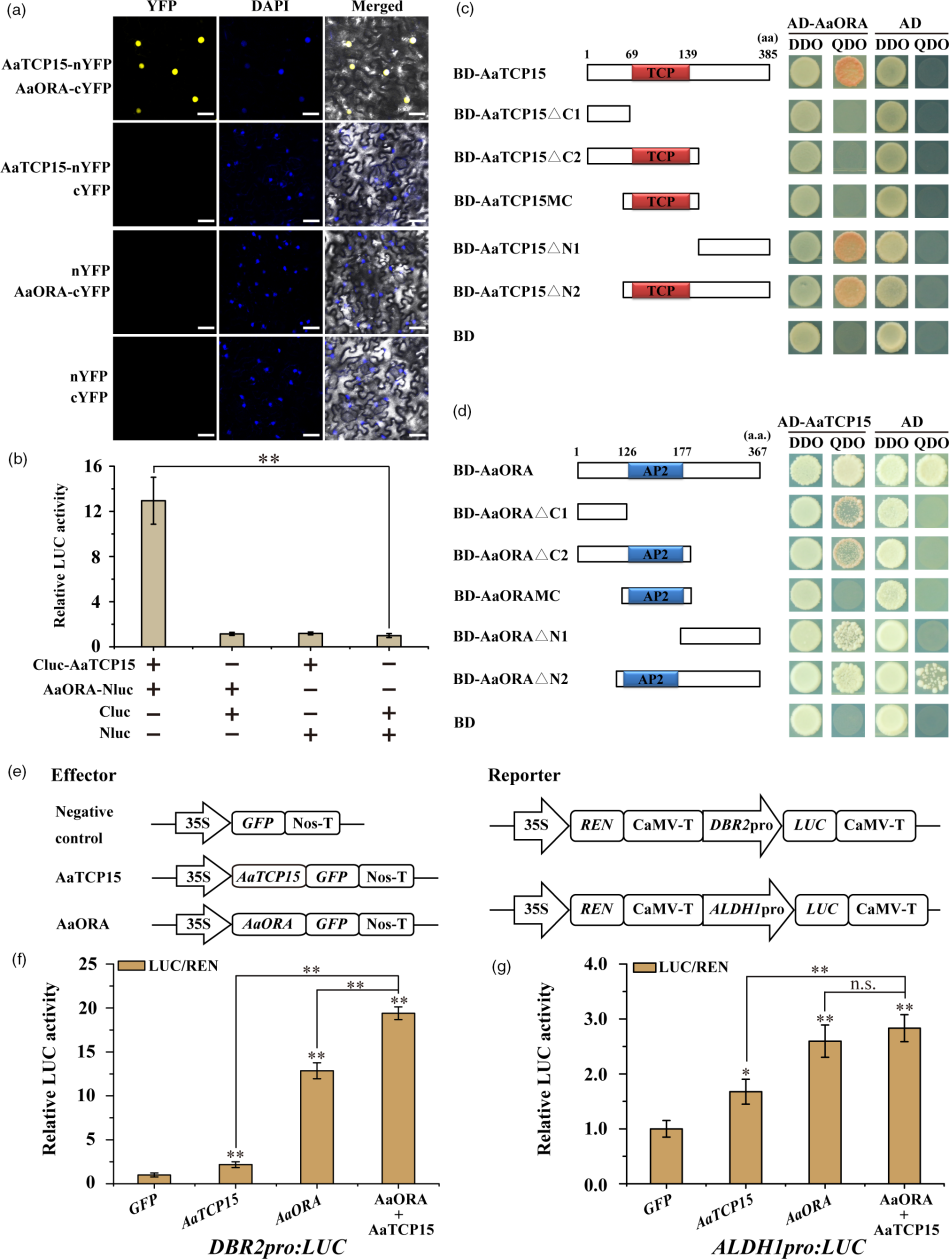
AaORA interacts with and enhances the transactivation activity of AaTCP15 on *DBR2* promoter. (a) Bimolecular fluorescence complementation (BiFC) analysis of the interaction between AaTCP15 and AaORA in *N*. *benthamiana* leaf cells. AaTCP15 was fused to the N‐terminal fragment of YFP (AaTCP15‐nYFP), and AaORA was fused to the C‐terminal fragment of YFP (AaORA‐cYFP). The nucleus was indicated by DAPI staining. Three independent transfection experiments were performed. Yellow fluorescence was detected using a confocal laser‐scanning microscope. Scale bar = 20 μm. (b) LUC complementation assay to detect the interaction between AaTCP15 and AaORA. AaTCP15 was fused to the C‐terminal fragment of LUC (Cluc‐AaTCP15), and AaORA was fused to the N‐terminal fragment of LUC (AaORA‐Nluc). LUC activity of Nluc and Cluc was set to 1. Three independent transfection experiments were performed. The data represent the means ± SD of 3 independent experiments. **P < 0.01, Student’s *t*‐test. (c) Y2H assays showing the interactions between AaORA and truncated versions of AaTCP15. Left, schematic representations of the truncated AaTCP15 protein used in this experiment. Numbers indicate the amino acid positions of the truncated AaTCP15 variants. The TCP domains are indicated by red boxes. Right, Y2H assays of protein interactions between AD‐AaORA and truncated versions of BD‐AaTCP15. (d) Y2H assays showing the interactions between AaTCP15 and truncated versions of AaORA. Left, schematic representations of the truncated AaORA protein used in this experiment. Numbers indicate the amino acid positions of the truncated AaORA variants. The AP2 domains are indicated by blue boxes. Right, Y2H assays of protein interactions between AD‐AaTCP15 and truncated versions of BD‐AaORA. The data represent three independent experiments, and representative results are shown. (e) A schematic representation of the constructs used in Dual‐LUC assays. (f, g) Activation of the *DBR2pro:LUC* (f) and *ALDH1pro:LUC* (g) by indicated combinations of AaORA and AaTCP15 proteins in *N. benthamiana* leaf cells, respectively. The GFP effector served as a negative control, and the LUC/REN ratios of GFP were set as 1. Three independent transfection experiments were performed. The reporter strain harbouring *DBR2pro:LUC* or *ALDH1pro:LUC* was mixed with the effector strains harbouring *35Spro:AaTCP15* and *35Spro:AaORA* at a ratio of 1 : 1 or 1 : 1 : 1. The data represent the means ± SD of three replicates from three independent experiments. *P < 0.05, **P < 0.01, Student’s *t*‐test, n.s., not significant.

In *Arabidopsis*, homologous TCP proteins may act as common components of the same regulatory pathway (Peng *et al.,* 2015). We previously reported that AaTCP14 together with AaORA formed a complex to regulate AN biosynthesis by their interaction (Ma *et al.,* 2018). Therefore, we hypothesized that AaTCP15 might also interact with AaORA. Indeed, in bimolecular fluorescence complementation (BiFC) assays, the AaTCP15 or AaORA fused with the N‐ or C‐terminus of YFP were transiently co‐expressed in *N. benthamiana* leaf cells by infiltration. The reconstituted YFP fluorescence signal was obviously observed in the nucleus, and merged with the signal of DAPI, a nuclear stain, supporting the interaction between AaTCP15 and AaORA (Figure 5a). Next, the interaction of AaTCP15 with AaORA was further corroborated by a LUC complementation experiment. When Cluc‐AaTCP15 and AaORA‐Nluc fusion proteins were co‐expressed in *N. benthamiana* leaf cells, strong relative LUC activity was detected, whereas those expressing Cluc‐AaTCP15 or AaORA‐Nluc alone showed low LUC activity (Figure 5b). Taken together, these results suggest that AaTCP15 interacts with AaORA in plant cells.

To further identify which regions of AaTCP15 or AaORA may be responsible for their interactions, we generated yeast two‐hybrid (Y2H) constructs in which the different truncated AaTCP15 or AaORA variants were fused with the BD (binding domain) or AD (activating domain) of the pGBKT7 or pGADT7 vector, respectively. Consistent with the above results, AaTCP15 interacted with AaORA (Figure 5c). Furthermore, domain deletion analysis revealed that the C‐terminal region of AaTCP15 (AaTCP15ΔN1) alone was able to interact with AaORA, but the truncated versions of AaTCP15 that lacked the C‐terminal domain (AaTCP15ΔC1 and AaTCP15ΔC2) or TCP domain (AaTCP15MC) could not (Figure 5c). Additionally, we found that either the N‐terminal (AaORAΔC1) or C‐terminal (AaORAΔN1) domain of AaORA alone was able to interact with AaTCP15 (Figure 5d). Thus, these results suggested that the C‐terminal region of AaTCP15 and both of the N‐ and C‐terminal regions of AaORA are important for the AaTCP15‐AaORA interaction.

It was previously shown that the transactivation activity of TCP proteins was modulated by their interacting‐partner (Ma *et al.,* 2018; Sun *et al.,* 2019). Here, AaTCP15 and AaORA were involved in positively regulating the transcripts of AN biosynthetic genes. Thus, we next examined how the interaction between AaTCP15 and AaORA affected the transactivation activity of AaTCP15 on its target genes. We performed Dual‐LUC assays in *N. benthamiana* leaves using the effector constructs (*35S:GFP*, *35S:AaTCP15*, and *35S:AaORA*) and the reporter plasmid (*DBR2pro*:*LUC* or *ALDH1pro*:*LUC*, see Figure 5e). The co‐expression of *AaTCP15* or *AaORA* alone with the reporter plasmid significantly increased *LUC* expression driven by *DBR2* or *ALDH1* promoters compared to the GFP control (Figure 5f,g). Most importantly, the *LUC* expression was further elevated when *AaTCP15* and *AaORA* were simultaneously co‐expressed compared to those of *AaTCP15* or *AaORA* expressed alone (Figure 5f,g). The activity of *DBR2* promoter, rather than *ALDH1* promoter, which was enhanced by co‐expressing *AaTCP15* and *AaORA*, reached a significant level (*P* < 0.01) compared to *AaTCP15* or *AaORA* expressed alone (Figure 5f,g). Taken together, these results suggested that AaORA cooperatively enhanced the transactivation activity of AaTCP15 in preferentially regulating *DBR2* expression by their interaction.

### **The JA**‐ **and ABA**‐**responsive TF AaGSW1 directly activates *AaTCP15* expression to regulate AN biosynthesis**

**Figure 6:**
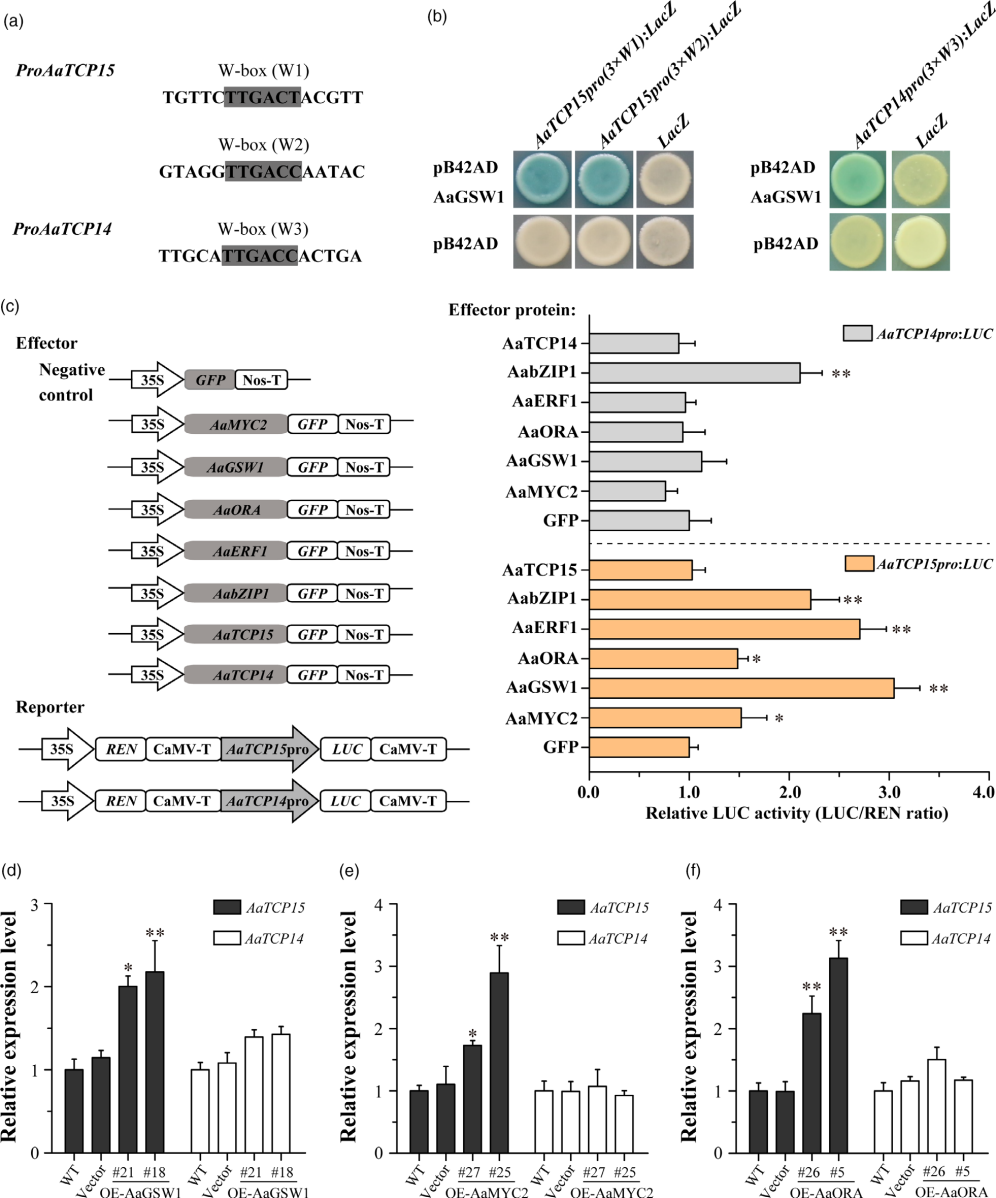
AaGSW1 directly and positively regulates the expression of *AaTCP15* rather than *AaTCP14*. (a) The fragments of *AaTCP15* and *AaTCP14* promoters containing the intact W‐box. The W‐box motif sequences of W1, W2 and W3 are shown as grey boxes. (b) Yeast one‐hybrid (Y1H) assays showing that AaGSW1 binds to the W1 and W2 motif of *AaTCP15* promoter, and W3 motif of the *AaTCP14* promoter. Three tandem repeats of W1, W2 and W3 motifs were used as baits. Transformed yeast cells were grown on selective medium SD/‐Trp/‐Ura containing 20 mg/L X‐gal, and pictures were taken after 4 days of incubation at 30 °C. Blue plaques indicate protein‐DNA interactions. The Y1H assays were repeated three times, and representative results are shown. (c) Left, schematic diagrams of the effector and reporter plasmids used in Dual‐LUC assays. REN, *Renilla* luciferase. LUC, firefly luciferase. Right, Dual‐LUC assay in *N. benthamiana* leaf cells using the constructs shown at Left. The GFP effector was used as a negative control, and the LUC/REN ratios of GFP were set as 1. Three independent transfection experiments were performed. The data represent the means ± SD of three replicates from three independent experiments. *P < 0.05, **P < 0.01, Student’s *t*‐test. (d‐f) Expression levels of *AaTCP15* and *AaTCP14* in the leaves of different *A. annua AaGSW1* (d), *AaMYC2* (e) and *AaORA* (f) overexpression lines, and plants transformed with the empty vector (labelled as Vector) and WT. *AaActin* was used as the internal control. The data represent the means ± SD of three replicates from three cutting propagations. *P < 0.05, **P < 0.01, Student’s *t*‐test.

Our current report demonstrated that the *AaTCP15* transcript is induced after JA or ABA treatment (Figure 2e), and the suppression of *AaTCP15* expression significantly reduced AN content and attenuated the JA‐ or ABA‐induced AN accumulation (Figures 3 and S5). These observations supported that AaTCP15 is a key positive regulator in AN biosynthesis, and JA and ABA promote AN biosynthesis by activating downstream *AaTCP15* expression in *A. annua*. To better identify the upstream regulators that link JA or ABA signalling and lead to the activation of *AaTCP15*, we first analysed the cis‐acting regulatory elements in the promoter of *AaTCP15* using PlantCARE tool (http://bioinformatics.psb.ugent.be/webtools/plantcare/html/). Apart from the common light, hormonal (i.e. ABA and MeJA) and abiotic stress responsiveness elements (Figure S6), two or one conserved W‐box motif known to be bound by WRKY TFs (Chen *et al.,* 2017) were also found in *AaTCP15* or its homologous gene *AaTCP14* promoter, respectively (Figure 6a). This suggested that *AaTCP15* or *AaTCP14* might be regulated by WRKY family genes.

In this context, we tested whether the earlier reported JA and ABA dual‐responsive WRKY TF AaGSW1 (Chen *et al.,* 2017), which acted at the nexus of JA and ABA signalling to positively regulate JA‐ and ABA‐induced AN biosynthesis, could bind to *AaTCP15* or *AaTCP14* promoters through Y1H assays. Results found that AaGSW1 directly bound to the *W1* and *W2* motifs in the *AaTCP15* promoter or *W3* motif in the *AaTCP14* promoter (Figure 6a,b). Then, we investigated whether AaGSW1 could activate *AaTCP15*/*14* expression by employing Dual‐LUC assays in *N. benthamiana* leaves and found that AaGSW1 significantly enhanced *AaTCP15* but not *AaTCP14* promoter activity (Figure 6c). To further verify this finding, we screened two independent *AaGSW1* transgenic lines (OE‐AaGSW1‐18, 21) in which the expression of *AaGSW1* and the AN content were significantly increased compared to Vector controls (Figure S7a,d). Expression of *AaTCP15* rather than *AaTCP14* was significantly increased in *AaGSW1* transgenic lines (Figure 6d), which was in accordance with the Dual‐LUC assays (Figure 6c). These results revealed that JA and ABA promoted *AaTCP15* but not *AaTCP14* expression directly by JA and ABA dual‐responsive TF AaGSW1, and AaGSW1 along with AaTCP15 may form a JA and ABA stepwise responsive AaGSW1‐AaTCP15 transcriptional regulatory cascade to control AN biosynthesis.

In addition, our Dual‐LUC assays also showed that several JA‐responsive TFs, AaMYC2, AaORA, AaERF1 and ABA‐responsive TF AabZIP1, which positively promote AN biosynthesis by JA and ABA, enhanced the *AaTCP15* promoter activity, whereas AaTCP15 itself had a negligible effect on its own promoter activity (Figure 6c). In a parallel assay, we found that although AaTCP14 is homologous with AaTCP15 (Figure 1a), only AabZIP1 but not AaMYC2, AaORA, AaERF1 or AaTCP14 itself could enhance the *AaTCP14* promoter activity (Figure 6c). This finding was consistent with the result that expression of *AaTCP14* was induced under ABA treatment (Figure S8), implying that JA and ABA signalling may regulate *AaTCP15* or *AaTCP14* expression through a distinct or partially similar upstream regulator in *A. annua*. Additionally, in accordance with the above results (Figure 6c), we found that the *AaTCP15*, but not the *AaTCP14*, transcript was significantly up‐regulated in *AaMYC2* (OE‐AaMYC2‐27, 25) or *AaORA* (OE‐AaORA‐26, 5) overexpressed *A. annua* lines (Figures 6e,f and S7b,c), in which the AN content is significantly higher compared to WT or Vector controls (Figure S7e,f). Taken together, these results implied that apart from the JA and ABA dual‐responsive TF AaGSW1, the multiple JA or ABA‐responsive TFs could also activate *AaTCP15* expression to control AN content in *A. annua*.

## Discussion

**Figure 7:**
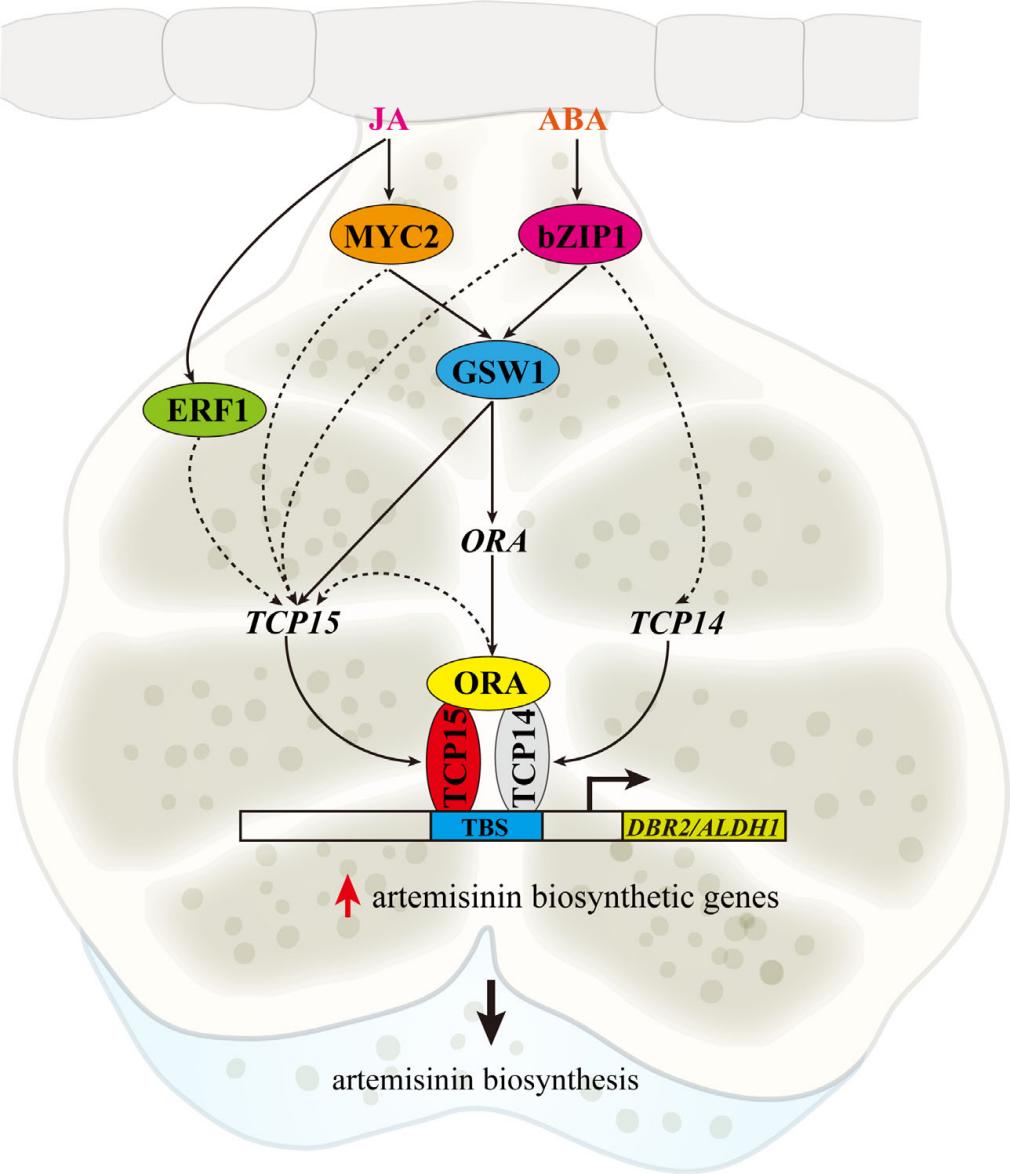
Proposed working model depicting how the AaTCP15‐related module regulates artemisinin biosynthesis in response to JA and ABA signalling. AaMYC2, AaERF1 and AabZIP1 transcription factors are positive regulators of artemisinin (AN) biosynthesis, which are positively activated by JA or ABA (Shen *et al.,* 2016; Yu *et al.,* 2012; Zhang *et al.,* 2015). AaMYC2 and AabZIP1 directly activate the expression of their common target gene *AaGSW1*, a positive regulator of AN biosynthesis through linking JA and ABA signalling (Chen *et al.,* 2017). AaGSW1 binds to and activates the *AaTCP15* promoter except for the reported *AaORA* promoter, which may facilitate the formation of AaTCP15‐AaORA regulatory module by their interaction. In addition, AaMYC2, AabZIP1 and AaERF1 may be also involved in activating *AaTCP15* expression. AaTCP15 enhances AN biosynthesis through directly binding to and activating the target genes *DBR2* and *ALDH1* promoters. AaORA interacts with AaTCP15 to enhance its transactivation activity on the AaTCP15 target gene and also promotes *AaTCP15* expression, thereby leading to the synergistic activation of AN biosynthetic genes, and finally promoting AN biosynthesis through responding to upstream JA and ABA signalling. AabZIP1 is also involved in activating *AaTCP14* expression, and the complex of AaTCP15‐AaORA and AaTCP14‐AaORA (Ma *et al.,* 2018) may be involved in a common module to regulate artemisinin content. TBS, TCP binding site. Solid arrow, direct regulation; Red arrow, up‐regulation. Dashed line arrow, hypothetical direct links. The background image is the model of a glandular trichome of *A. annua*.

Artemisinin (AN) is a sesquiterpene lactone endoperoxide derived from *A*. *annua*. AN is not only effective against malaria, but also has great application potential in treating lupus‐related nephritis, viral infections, schistosomiasis, tuberculosis, cancer and diabetes (Crespo‐Ortiz and Wei, 2012; Efferth *et al.,* 2008; Li *et al.,* 2017, 2006; Liu *et al.,* 2011; Tin *et al.,* 2012; Zheng *et al.,* 2017). It has been recently reported that AN biosynthesis is controlled by external stimuli. Of these, JA and ABA have attracted extensive attention due to their vital roles in promoting AN biosynthesis (Jing *et al.,* 2009; Maes *et al.,* 2011). Notably, although previous studies have demonstrated that JA and ABA governed AN through activating downstream TFs including WRKY, bZIP, bHLH and ERF (Chen *et al.,* 2017; Ji *et al.,* 2014; Shen *et al.,* 2016; Yu *et al.,* 2012; Zhang *et al.,* 2015), the specific molecular mechanism linking JA and ABA signalling with AN biosynthesis by activating the downstream TFs associated regulatory network as‐yet remains enigmatic. In this study, we found that AaTCP15 had an important role in positively promoting JA and ABA‐induced AN biosynthesis. Further, AaORA, an activator of AN biosynthesis, enhanced the transactivation activity of AaTCP15 on its target gene by direct interaction with AaTCP15, and simultaneously activated *AaTCP15* expression. In addition, the *AaTCP15* transcript was activated by JA and ABA through employing multiple downstream TFs involved in the JA‐ or ABA‐governed AN biosynthesis process. Of these, AaGSW1, a positive regulator that acts at the nexus of JA and ABA signalling to activate AN biosynthesis, activated *AaTCP15* expression by directly binding to its promoter. Therefore, our study proposes an AaGSW1‐AaTCP15/AaORA transcriptional regulatory module that positively regulates AN biosynthesis through simultaneously responding to JA and ABA signalling in *A. annua* (Figure 7).

Up till now, only a few studies reported the upstream regulatory pathway of class II TCP factors. For instance, the C_2_H_2_ zinc finger gene *RABBIT EARS* (*RBE*) repressed the transcription of *TCP4* during *Arabidopsis* petal development (Li *et al.,* 2016). In *Arabidopsis*, *miR319* targeted *TCP2*, *TCP3*, *TCP4*, *TCP10* and *TCP24* regulated leaf morphogenesis (Palatnik *et al.,* 2003). However, upstream regulators of the class I TCP family are still unknown. Strikingly, we found that the expression level of *AaTCP15* was induced by both JA and ABA treatments at later time points (Figure 2e), and *AaTCP15*‐antisense *A. annua* (Anti‐AaTCP15) had lower JA‐ or ABA‐induced AN accumulation (Figure S5). These results suggest that there might be a novel mechanism upstream of AaTCP15 involved in JA and ABA‐mediated AN biosynthesis. JA and ABA‐responsive AaMYC2, AaGSW1, AaERF1 and AabZIP1 are reported to positively regulate AN biosynthesis through activating structural gene promoters (Chen *et al.,* 2017; Shen *et al.,* 2016; Yu *et al.,* 2012; Zhang *et al.,* 2015). Interestingly, we found that these TFs all activated *AaTCP15* promoter, and they might form a novel AaMYC2/AabZIP1‐AaGSW1‐AaTCP15 transcriptional cascade (Figure 7). It has been reported that AaORA activated all four structural genes (*ADS*, *CYP71AV1*, *DBR2* and *ALDH1*) to modulate AN biosynthesis (Lu *et al.,* 2013; Ma *et al.,* 2018), and we found that AaORA modulated AN biosynthesis through AaTCP15 at multiple layers. On the one hand, AaORA increased the transcriptional activation activity of AaTCP15 on *DBR2* promoter through forming an AaORA‐AaTCP15 complex at the protein level. On the other hand, AaORA activated the expression of *AaTCP15* at the transcriptional level. Thus, AaORA is a key regulator in AN biosynthesis through various mechanisms. By contrast, JA‐responsive transcription factors AaMYC2, AaGSW1, AaERF1 and AaORA could not activate *AaTCP14* promoter (Figure 6c). Although AaGSW1 could bind to *AaTCP14* promoter in the Y1H assay (Figure 6b), it could not activate the expression of *AaTCP14* (Figure 6c), suggesting that AaGSW1 may need another activator to coordinately modulate *AaTCP14* expression. Hence, the AaGSW1‐AaTCP15/AaORA transcriptional cascade is specific to AaTCP15 but not AaTCP14, suggesting different molecular mechanisms between AaTCP15 and AaTCP14 in regulation of AN biosynthesis. These results broaden the network of JA and ABA regulation of specialized metabolites in plants. In addition, previous reports indicated that co‐expression of multiple genes than single gene in AN biosynthesis by metabolic engineering is an effective approach to increase AN content (Chen *et al.,* 2013; Shi *et al.,* 2017). In view of the components of AaGSW1‐AaTCP15/AaORA regulatory cascade could control the expression of different AN biosynthetic genes, co‐overexpression of *AaGSW1*, *AaTCP15* and *AaORA* may be a potential and promising strategy to elevate the production of AN in the future in engineered *A. annua* plants.

AaTCP15 and AaTCP14 are homologous genes that belong to the class I TCP family, which can work synergistically or function alone. TCP15 and TCP14 interacted with SPINDLY to facilitate cytokinin responses in leaves and flowers in *Arabidopsis* (Steiner *et al.,* 2012a), and they co‐regulated cytokinin‐induced branching and meristematic activity in tomato (Steiner *et al.,* 2012b). Moreover, in *Arabidopsis*, TCP15 and TCP14 modulated internode length and leaf shape via cell division (Kieffer *et al.,* 2011), regulated gynoecium development through balancing auxin and cytokinin responses (Lucero *et al.,* 2015) and controlled endoreduplication together with DA1, DAR1 and DAR2 (Peng *et al.,* 2015). Likewise, we found that AaTCP15 directly bound and activated *DBR2* and *ALDH1* promoters, similar to AaTCP14. Although *AaTCP15* and *AaTCP14* were both expressed in GSTs and TSTs, they showed different expression pattern in GSTs. *AaTCP15* showed strong expression at the end of two cells of GST (Figure 2d), while *AaTCP14* was expressed in all ten cells of GST (Ma *et al.,* 2018), indicating that they may have different regulatory mechanisms in AN biosynthesis.

Interestingly, in OE‐AaTCP15 transgenic *A. annua*, the AN content increased but DHAA content decreased (Figures 3 and S4), whereas in OE‐AaTCP14 transgenic *A. annua*, both AN and DHAA content increased (Ma *et al.,* 2018). This indicates that in OE‐AaTCP15 transgenic *A. annua*, the metabolic flux from artemisinic aldehyde to AN may remain constant, and the increased AN may be due to the enhanced efficiency from DHAA to AN, the last step in AN biosynthesis. However, in OE‐AaTCP14 transgenic *A. annua*, the metabolic flux from artemisinic aldehyde to AN may increase, thus increasing both AN and DHAA content. DHAA is considered the direct substrate of AN (Zhu *et al.,* 2014) and is then converted to AN through an oxidation reaction, a process that requires species specific enzymes to obtain specialized metabolites. In rice, the short‐chain alcohol dehydrogenase/reductases including OsSDR110C‐MS2 and OsSDR110C‐MS1 (OsMAS) catalysed the final oxidation reaction in the biosynthesis of rice diterpenoid, momilactone A (Murphy and Zerbe, 2020). Moreover, in *Mentha spicata*, carveol dehydrogenase (CDH) catalysed the final oxidation reaction in the biosynthesis of carvone (Ahkami *et al.,* 2015). The last step of AN biosynthesis is still mysterious due to the unidentified enzyme responsible for the conversion from DHAA to AN, thus *AaTCP15* might be involved in the activation of the unknown enzyme activity, which finally leads to decreased or increased DHAA content in OE‐AaTCP15 or in Anti‐AaTCP15 transgenic *A. annua*, respectively. Correspondingly, elevated activity of this unknown enzyme might also be one of the reasons for the AN increase in OE‐AaTCP15 or decrease in Anti‐AaTCP15 transgenic *A. annua*.

Moreover, like AaTCP14, AaTCP15 also formed the AaTCP15‐AaORA complex by interaction (Figure 5), but unlike AaTCP14, which only interacted with the C‐terminus of AaORA (AaORAΔN1; Ma *et al.,* 2018), AaTCP15 interacted with both the C‐terminus (AaORAΔN1) and N‐terminus of AaORA (AaORAΔC1; Figure 5d). Because of these different interaction regions, AaORA might have different synergistic activation effects between AaTCP15 and AaTCP14: the AaTCP15‐AaORA complex can only synergistically activate *DBR2* promoter (Figure 5), but the AaTCP14‐AaORA complex can synergistically activate both *DBR2* and *ALDH1* promoters (Ma *et al.,* 2018). Taken together, AaTCP15 and AaTCP14 may have some redundancy in regulating AN biosynthesis, but they have predominantly independent roles in AN biosynthesis. Therefore, it is of great significance to unravel the distinct roles of other TCP genes to modulate AN biosynthesis.

In conclusion, we found that AaTCP15 activates AN biosynthesis through JA and ABA signalling pathways; hence, AaTCP15 is a key point in these two pathways to modulate AN biosynthesis. In this study, the molecular network for JA and ABA regulation of AN biosynthesis was expanded, laying a firm foundation for cultivating varieties with high AN content by using transcriptional strategies and providing a reference for the regulation of specialized metabolites in other plants.

## Methods

### Plant materials and treatments

The high AN content *A. annua*, ‘Huhao 1’, which comes from Chongqing and was selected for several years in Shanghai, was used for all *A. annua* related assays (Shen *et al.,* 2016). *A. annua* and *N. benthamiana* plants were grown in light chambers at 23 ± 2 °C under a 16‐h light/8‐h dark photoperiod.

For MeJA, ABA and salt treatments, 10‐day‐old *A. annua* seedlings were sprayed with 100 μm MeJA (Sigma, St. Louis, MO, USA), 100 μm ABA (Sigma) and 150 mm NaCl (Sigma). For the mock treatment, *A. annua* seedlings were sprayed with 0.1% ethanol. The leaves of *A. annua* seedlings in the same position were harvested at 0, 0.5, 1, 3, 6, 9, 12 and 24 h after treatment, respectively. To analyse AN content in *AaTCP15*‐antisense (Anti‐AaTCP15) plants after MeJA or ABA treatment, 2‐month‐old cutting propagations of Anti‐AaTCP15, wild‐type (WT) and Vector controls (*A. annua* plants transformed with the empty vector, labelled as Vector) were sprayed with 100 μm MeJA, 50 μm ABA or 0.05% ethanol (Mock treatment) and sampled at 72 h for AN extraction.

### RNA extraction and quantitative real‐time PCR (qRT‐PCR)

Different leaves (leaf 1, leaf 2, leaf 3, leaf 4, leaf 5, leaf 6, leaf 7, leaf 8 and leaf 9, labelled in Figure 2a) of 3‐month‐old *A. annua* and different tissues (roots, stems, flowers, shoots, buds 0, buds 1, young leaves, old leaves and trichomes) of 4‐month‐old *A. annua* were collected to analyse the expression of the indicated genes. To detect the expression of *AaTCP15*, *AaADS*, *AaCYP71AV1*, *AaDBR2* and *AaALDH1* in *AaTCP15* overexpression and antisense lines, and the expression of *AaTCP15* or *AaTCP14* in *AaMYC2*, *AaGSW1* or *AaORA* overexpression lines, the leaves of 3‐month‐old WT plants, Vector controls and the aforementioned transgenic plants were collected. Total RNA of the above samples was extracted using the RNAprep pure Plant Kit (TianGen Biotech,Shanghai, China). cDNA was synthesized from 1.0 μg total RNA using the PrimeScript 1st Strand cDNA Synthesis Kit (Takara, Japan) according to the manufacturer’s instructions. The qRT‐PCR analysis was performed as previously reported (Lu *et al.,* 2013) using the cDNA templates gained from the above indicated samples. All PCR reactions were conducted three times in independent experiments. The qRT‐PCR results were calculated by normalization to *AaActin*. Primers are listed in Table S1.

### *β‐glucuronidase* (*GUS*) expression in *1391Z‐proTCP15‐GUS* transgenic *A. annua* plants

The 2104 bp upstream region of the start codon of *AaTCP15* was amplified with specific primers (Table S1) from the *A. annua* genomic DNA library and inserted into the pCAMBIA‐1391Z vector to construct *1391Z‐proTCP15‐GUS* recombinant vector. Subsequently, the *Agrobacterium tumefaciens* strain EHA105 harbouring *1391Z‐proTCP15‐GUS* or *1391Z‐GUS* plasmids were introduced to *A. annua* leaves to obtain transgenic *A. annua* plants as previously reported (Zhang *et al.,* 2009). Histochemical staining for GUS activity in transgenic plants was conducted as previously described (Ma *et al.,* 2018). *A. annua* plants transformed with *1391Z‐GUS* empty vector (control plants) were processed in parallel as negative controls.

### Subcellular localization of AaTCP15

The GV3101 strains harbouring pHB‐AaTCP15‐YFP or pHB‐YFP vectors were transformed into 5‐week‐old *N. benthamiana* leaves for subcellular localization experiments. YFP fluorescence was observed at 60–72 h after infiltration by confocal laser microscopy (Leica TCS SP5‐II). Nuclei were stained with 4′, 6‐diamidino‐2‐phenylindole (DAPI, Sigma). Three biological repeats were performed to verify these results.

### Plant transformation and phenotype analysis

The overexpression constructs pCAMBIA1300‐AaTCP15‐GFP, pHB‐AaMYC2 (Shen *et al.,* 2016), pCYP‐AaGSW1 (Chen *et al.,* 2017), pCAMBIA1300‐AaORA‐GFP, and the antisense construct pCAMBIA1300‐Anti‐AaTCP15 were transferred into *A. tumefaciens* strain EHA105 and then used to transform *A. annua* as previously described (Zhang *et al.,* 2009). Briefly, the sterilized *A. annua* seeds were placed on MS_0_ medium and then cultured in a light chamber at 25 ± 1 °C under a 16‐h light/8‐h dark photoperiod. After 14 days, the leaves of germinated seedlings were collected and cut into 0.5 cm diameter discs and used as explants that were co‐cultivated with *A. tumefaciens* strain EHA105 containing the above construct at 25 °C for 3 days. After this, the transformed explants were selected in the selective medium MS_1_ (MS_0_ + 2.5 mg/L N_6_‐benzoyladenine + 0.3 mg/L naphthalene‐acetic acid) containing 50 mg/L hygromycin; the resistant plantlets were regenerated and sub‐cultured twice and then transferred into MS_2_ (½ MS_0_ + 250 mg/L carbenicillin) medium for root growth. After 1 month, the rooted plantlets were selected and planted in soil in a light chamber at 23 ± 2 °C under a 16‐h light/8‐h dark photoperiod. The phenotype of Vector control plants (labelled as Vector), *AaTCP15*‐overexpression (OE‐AaTCP15) and *AaTCP15*‐antisense (Anti‐AaTCP15) lines were observed at two and a half months in a greenhouse under normal conditions.

### Measurement of artemisinin (AN) and DHAA content

Leaves of 3‐month‐old OE‐AaTCP15 and Anti‐AaTCP15 *A. annua* plants, Vector controls and WT plants grown in the greenhouse were harvested and dried at 50 °C for 2 days and then ground to powder. The extraction and measurement methods were conducted as previously described (Ma *et al.,* 2018).

### **Dual**‐**luciferase assay**

The full‐length coding sequences of *AaTCP15*, *AaMYC2*, *AaGSW1*, *AaORA*, *AaERF1* and *AabZIP1* were amplified and inserted into pCAMBIA1300‐GFP vectors (effectors), and the promoter regions upstream of the start codon of *AaADS* (~1.9 kb), *AaCYP71AV1* (~1.2 kb), *AaDBR2* (~2.3 kb), *AaALDH1* (~2.3 kb), *AaTCP15* (~2.1 kb) and *AaTCP14* (~1.9 kb) were ligated into pGREENII0800‐LUC vector (reporters). The *Renilla* luciferase (REN) driven by the cauliflower mosaic virus (CaMV) 35S promoter in pGREENII0800‐LUC was used as an internal control. Empty pCAMBIA1300‐GFP was used as the negative control for the effector. Infiltration and detection were performed as described previously (Ma *et al.,* 2018). The ratio of firefly luciferase to *Renilla* luciferase represents the relative activity of the promoter. All experiments were repeated three times for each combination. Primers are listed in Table S1.

### Yeast one‐hybrid assay

The full‐length coding sequences of *AaTCP15* and *AaGSW1* were amplified and inserted into pB42AD vector. Three tandem copies of the *D* motif from *DBR2* promoter, *A* motif from *ALDH1* promoter, *W1* and *W2* motifs (W‐box, Figure 6a) from *AaTCP15* promoter, and *W3* motif (W‐box, Figure 6a) from *AaTCP14* promoter were separately ligated into pLacZ vector. Y1H assays were conducted as described previously (Ma *et al.,* 2018). All primers are listed in Table S1.

### Electrophoretic mobility shift assay

For protein expression and purification, the full‐length coding sequence of *AaTCP15* was cloned into pCold™‐TF (trigger factor) vector (Takara, Japan) to produce His‐tagged fusion protein. The expression and purification of His‐TF protein and His‐AaTCP15 fusion proteins were performed as described previously (Ma *et al.,* 2018).

EMSA assays were conducted as described previously (Ma *et al.,* 2018). The Dq, Dq‐mutated, Aq and Aq‐mutated DNA probes from the *DBR2* or *ALDH1* promoters were synthesized by Sangon (Shanghai, China). The mutated probes were designed in accordance with a previous report (Pruneda‐Paz *et al.,* 2009). The primers and probes used in the EMSA assays are listed in Table S1.

### Bimolecular fluorescence complementation assay

The amplicons of *AaTCP15* or *AaORA* were ligated into pEarleyGate 201‐YN (N‐terminal of YFP) or pEarleyGate 202‐YC (C‐terminal of YFP), respectively. The resultant AaTCP15‐nYFP, AaORA‐cYFP vectors were transformed into *Agrobacterium* strains GV3101. The BiFC assays were conducted as previously described (Ma *et al.,* 2018; Shen *et al.,* 2016). Three independent experiments were conducted. The primers are listed in Table S1.

### Luciferase complementation assay

The LUC complementation assays were performed as previously described (Ma *et al.,* 2018). In brief, the amplicons of *AaTCP15* and *AaORA* were inserted into pCAMBIA‐Nluc or pCAMBIA‐Cluc vector, respectively. The yield Cluc‐AaTCP15 and AaORA‐Nluc vectors were transformed into *Agrobacterium* strain GV3101. The indicated combinations were co‐transformed into 5‐week‐old *N. benthamiana* leaves. Leaf discs were harvested 3 days later and ground to powder in liquid nitrogen. Subsequently, the relative LUC activities were measured by the Commercial Luciferase Kit according to the manufacturers’ instructions (Promega, Durham, NC, USA). The relative LUC activities of Nluc and Cluc were set as 1. The primers are listed in Table S1.

### Yeast two‐hybrid experiments

To test the physical interaction between AaTCP15 and AaORA, and map the regions responsible for this paired interaction, the full‐length coding sequences of *AaTCP15* and *AaORA* were cloned into pGADT7 (AD) or pGBKT7 (BD) (Takara, Japan), respectively, and their truncated sequences including *AaTCP15ΔC1*, *AaTCP15ΔC2*, *AaTCP15MC*, *AaTCP15ΔN1*, *AaTCP15ΔN2*, *AaORAΔC1*, *AaORAΔC2*, *AaORAMC*, *AaORAΔN1* and *AaORAΔN2* were amplified using specific primers (Table S1) and inserted into the bait vector BD. Various combinations were co‐transformed into yeast strain AH109 according to the manufacturer’s instructions (Takara, Japan), and bait‐only or prey‐only was tested with empty AD or BD as negative controls. The Y2H assays were carried out as previously reported (Ma *et al.,* 2018). All experiments were repeated three times with similar results.

### Accession Numbers

The sequences of all genes mentioned in this article are available in the NCBI database as follows: *AaTCP14* (MF770262), *AaTCP15* (MK770359), *AaORA* (JQ797708), *AaMYC2* (KP119607), *AaGSW1* (KX465128), *proADS* (DQ448294), *proCYP71AV1* (FJ870128), *proDBR2* (KC118523.1) and *proALDH1* (KC118525.1). All other data related to the conclusions in the paper can be found in the paper and/or the Supplemental Materials.

## Conflict of interest

The authors declare that they have no competing interests.

## Author contributions

K. X. T., Y. N. M. conceived of and supervised the research. Y. N. M., D. B. X. designed the experiments. Y. N. M., D. B. X., X. Y., Z. K. Y. W., and P. L. performed the experiments. S. I. K., X. Q. F., Q. S., Q. F. P., L. L., Z. Y. L., L. H. X., X. L. H., D. H., H. L. and X. F. S. analysed the data. Y. N. M., D. B. X. organized and wrote the manuscript. All authors read and approved the final manuscript.

## Supporting information


**Figure S1** A graphical representation of biosynthetic pathways and regulation of artemisinin in *Artemisia annua*.
**Figure S2** Phylogenetic tree showing the relationship of TCP transcription factors in *Artemisia annua*, *Arabidopsis* and *Gossypium raimondii*.
**Figure S3** Alignment of the protein sequences of TCP15 among *Artemisia annua*, *Arabidopsis* *thaliana* and *Gossypium raimondii*.
**Figure S4** Characterization of the content of DHAA and phenotype of *AaTCP15* transgenic plants.
**Figure S5** Artemisinin content in *AaTCP15*‐antisense plants under MeJA and ABA treatment.
**Figure S6** Analysis of the putative regulatory elements in the cloned *AaTCP15* promoter nucleotide sequence.
**Figure S7** Analysis of artemisinin contents in *AaGSW1*, *AaMYC2*, and *AaORA* overexpression *A. annua* plants.
**Figure S8** The expression pattern of *AaTCP14* under ABA treatment in *A. annua*.
**Table S1** List of primers used in this study
