# Supplementary material for: Jasmonate‐ and abscisic acid‐activated AaGSW1‐AaTCP15/AaORA transcriptional cascade promotes artemisinin biosynthesis in Artemisia annua
Source: Plant Biotechnol J. 2021 Feb 14;19(7):1412–28. doi: 10.1111/pbi.13561 (PMC8313134; doi:10.1111/pbi.13561)
Supplement: Supplementary file 1 — Figure S1 A graphical representation of biosynthetic pathways and regulation of artemisinin in Artemisia annua. Figure S2 Phylogenetic tree showing the relationship of TCP transcription factors in Artemisia annua, Arabidopsis and Gossypium raimondii. Figure S3 Alignment of the protein sequences of TCP15 among Artemisia annua, Arabidopsis thaliana and Gossypium raimondii. Figure S4 Characterization of the content of DHAA and phenotype of AaTCP15 transgenic plants. Figure S5 Artemisinin content in AaTCP15‐antisense plants under MeJA and ABA treatment. Figure S6 Analysis of the putative regulatory elements in the cloned AaTCP15 promoter nucleotide sequence. Figure S7 Analysis of artemisinin contents in AaGSW1, AaMYC2, and AaORA overexpression A. annua plants. Figure S8 The expression pattern of AaTCP14 under ABA treatment in A. annua. Table S1 List of primers used in this study [file PBI-19-1412-s001.docx]

**Supplemental Materials**


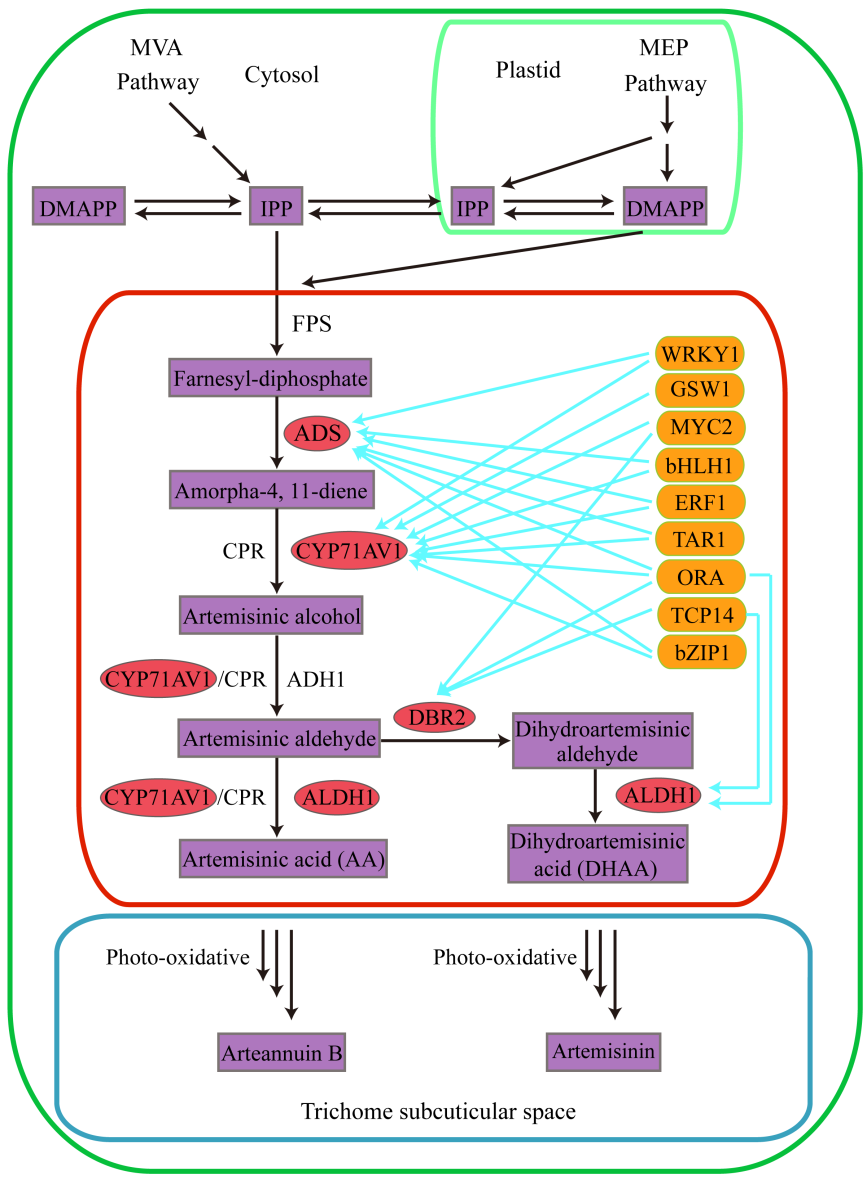


**Figure S1 A graphical representation of biosynthetic pathways and regulation of artemisinin in *Artemisia annua*.**

MVA, mevalonate; MEP, 2-C-methyl-D-erythritol 4-phosphate; IPP, isopentenyl diphosphate; DMAPP, dimethylallyl diphosphate; FPS, farnesyl diphosphatesynthase; ADS, amorpha-4,11-diene synthase; CYP71AV1, cytochrome P450 monooxygenase; CPR, cytochrome P450 reductase; ADH1, alcohol dehydrogenase 1; DBR2, artemisinic aldehyde Δ11(13) reductase; ALDH1, aldehyde dehydrogenase 1; WRKY1, WRKY transcription factor 1; GSW1, glandular trichome-specific WRKY1; MYC2, myelocytomatosis protein 2; bHLH1, basic helix-loop-helix (bHLH) transcription factor 1; ERF1, apetala2/ethylene-responsive factor (AP2/ERF) 1; TAR1, trichome and artemisinin regulator 1; ORA, octadecanoid-derivative responsive AP2-domain protein; TCP14, teosinte branched 1/cycloidea/proliferating cell factor 14; bZIP1, basic leucine zipper transcription factor 1.


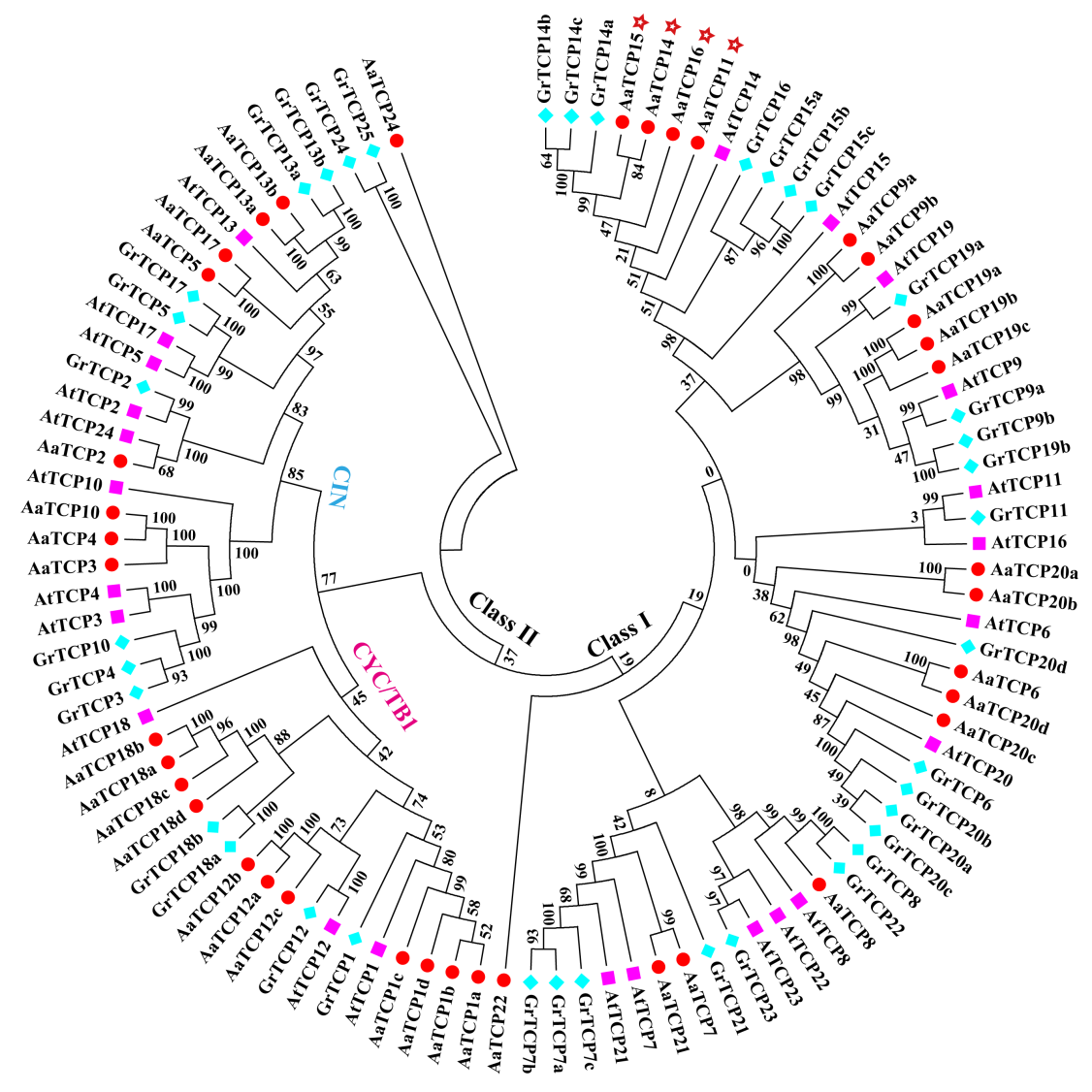


**Figure S2 Phylogenetic tree showing the relationship of TCP transcription factors in *Artemisia annua*, *Arabidopsis* and *Gossypium raimondii*.**

TCP sequences were aligned with Clustal W, and a neighbor-joining phylogenetic tree was constructed using MEGA. Bootstrap values indicate the percentage of 2,000 replicates. TCP proteins of *Artemisia annua*, *Arabidopsis thaliana* and *Gossypium raimondii* are indicated with red circles, pink boxes, and blue squares, respectively. The candidate AaTCP11, AaTCP14, AaTCP16 and AaTCP15 are marked with red stars. The amino acid sequences are named based on the species name.


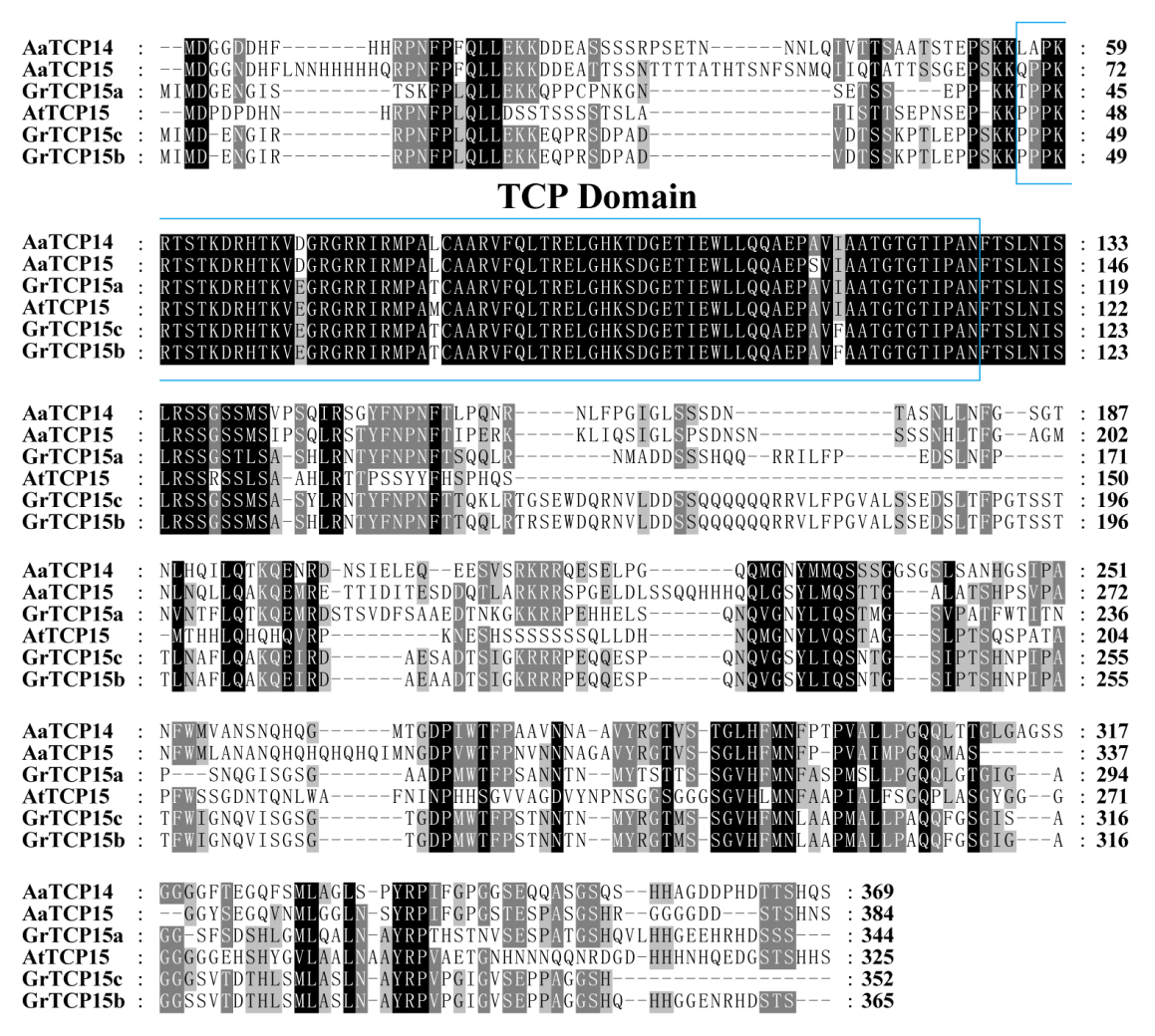


**Figure S3 Alignment of the protein sequences of TCP15 among *Artemisia annua*, *Arabidopsis* *thaliana* and *Gossypium raimondii*.**

The multiple sequence alignment of AaTCP15 and AaTCP14 in *Artemisia annua*, AtTCP15 in *Arabidopsis thaliana*, and GrTCP15a, GrTCP15b, GrTCP15c in *Gossypium raimondii* was generated by CLUSTALX2 using default parameters. Identical amino acids are shaded in black, conserved residues are shaded in dark grey and similar residues are shaded in light grey. The conserved TCP domains are outlined with a blue box.


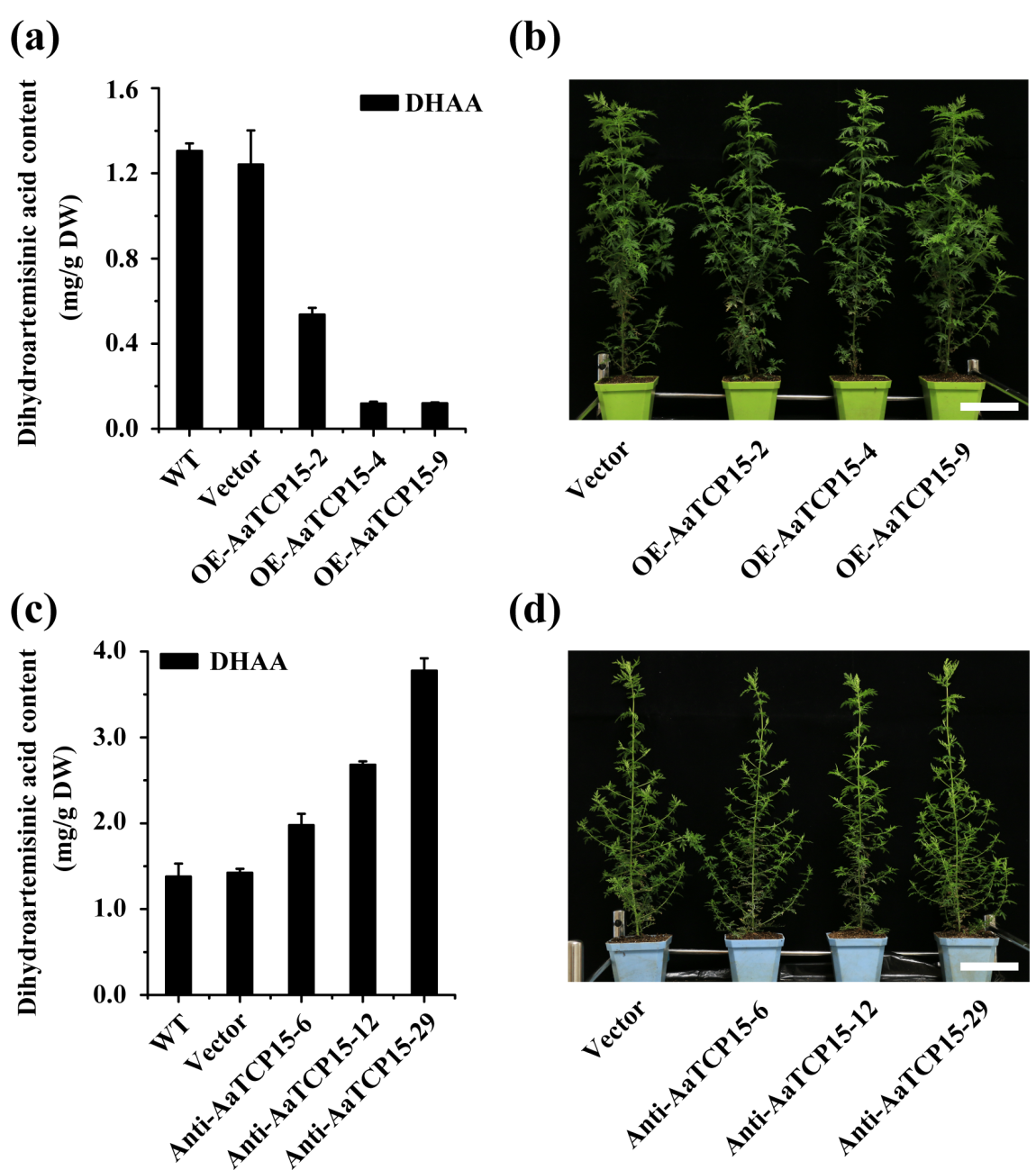


**Figure S4 Characterization of the content of DHAA and phenotype of *AaTCP15* transgenic plants.**

(a, c) HPLC analysis of dihydroartemisinic acid (DHAA) in the leaves of different *A. annua* *AaTCP15*-overexpression (OE-AaTCP15) lines (a) and *AaTCP15*-antisense (Anti-AaTCP15) lines (c), plants transformed with the empty vector (Vector) and wild-type (WT) plants. The data represent the means ± SD of three replicates from three cutting propagations. (b, d) The phenotypes of two and a half-month-old *A. annua* plants transformed with the empty vector (control plants, labeled as Vector), three independent OE-AaTCP15 lines (b) and Anti-AaTCP15 lines (d). Scale bar = 10 cm.


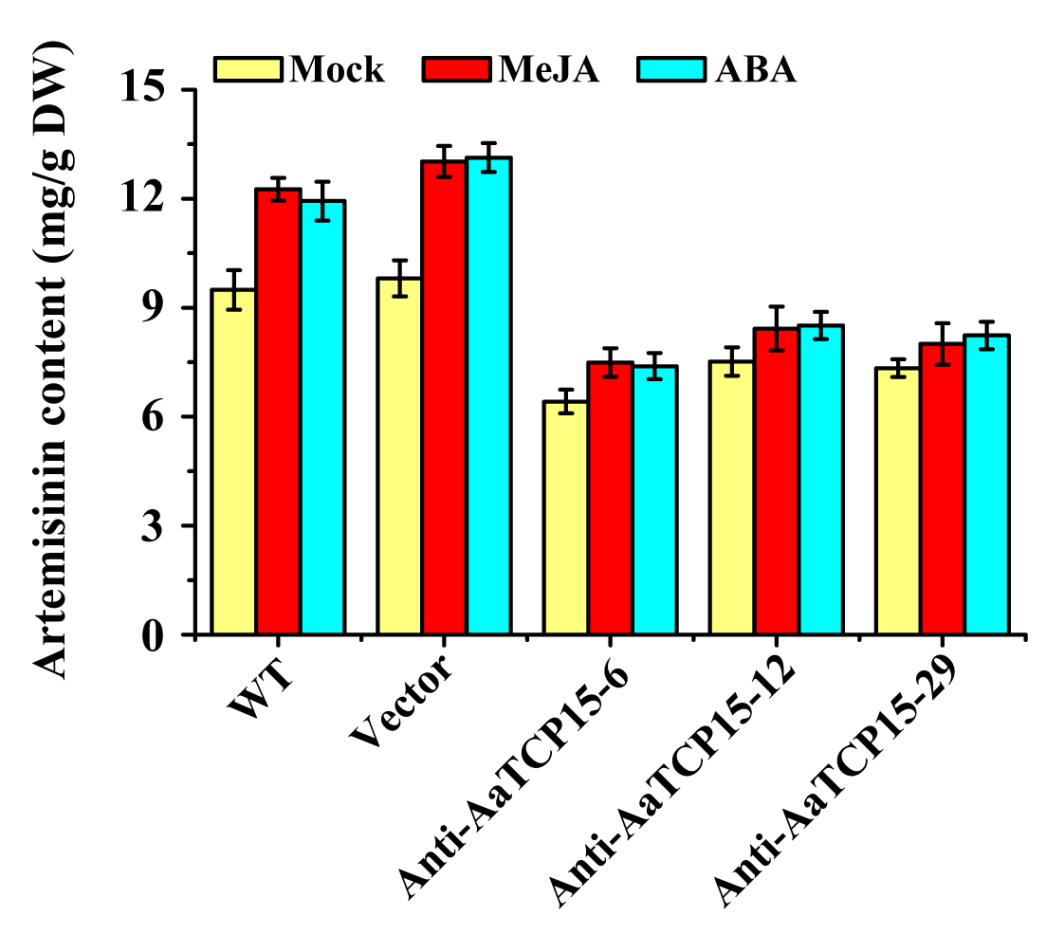


**Figure S5 Artemisinin content in *AaTCP15***-**antisense plants under MeJA and ABA treatment.**

Artemisinin content in *A. annua AaTCP15*-antisense (Anti-AaTCP15) lines, wild-type (WT) plants and plants transformed with the empty vector (control plants, labeled as Vector) was measured by HPLC after 72 h of treatment with 100 μM MeJA, 50 μM ABA or 0.05% ethanol (Mock). The data represent the means ± SD of three replicates from three cutting propagations.


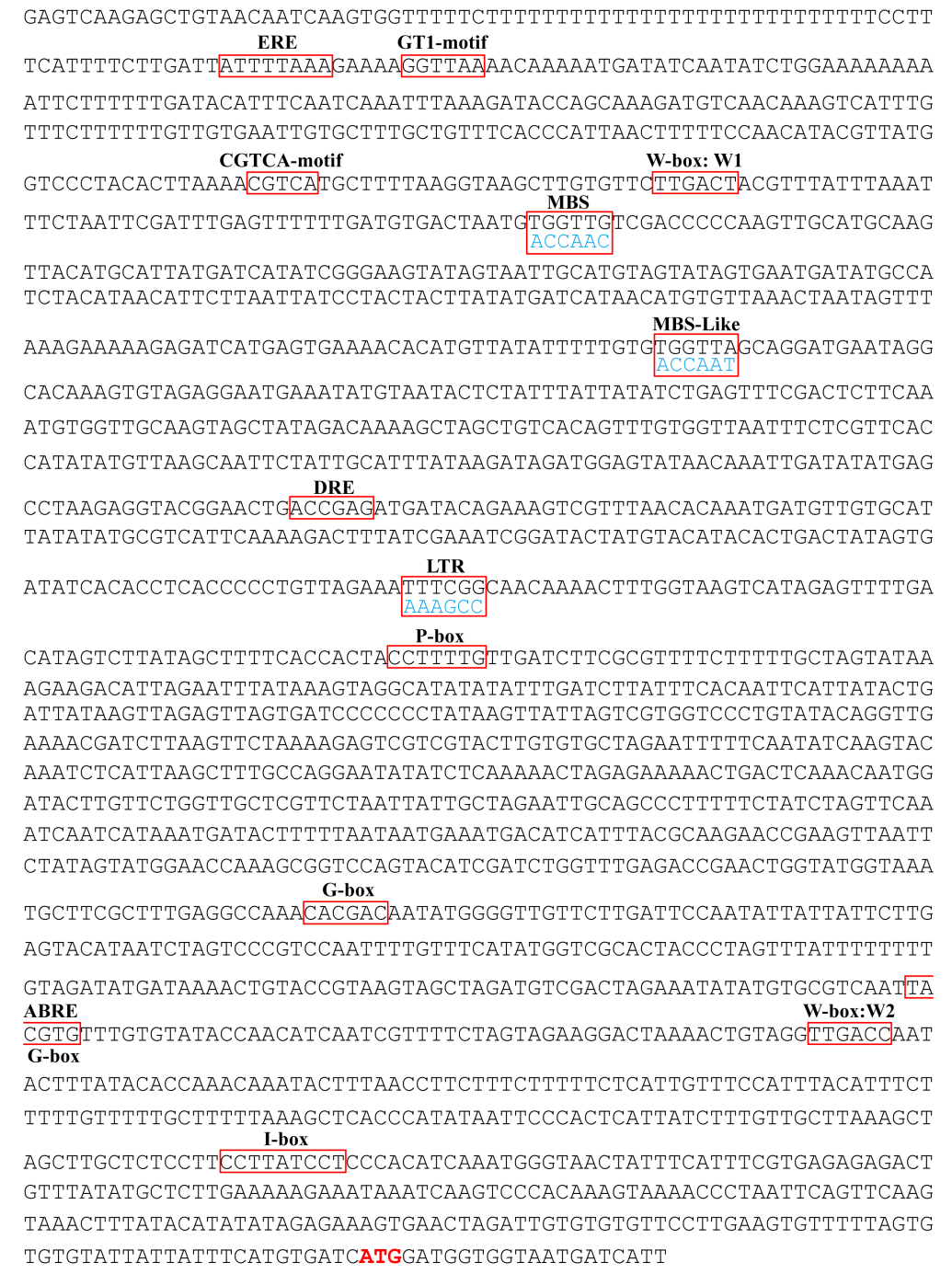


**Figure S6** **Analysis of the putative regulatory elements in the cloned *AaTCP15* promoter nucleotide sequence.**

The putative cis-regulatory elements are labeled with red rectangles, and the translational start site (ATG) is indicated with red and bold font. ERE, ethylene-responsive element. GT1-motif, G-box, and I-box, light-responsive element. CGTCA-motif, Methyl jasmonate (MeJA) responsive element. W-box (W1 and W2), WRKY transcription factor binding site. MBS and MBS-Like, MYB binding sites. DRE, drought responsive element. LTR, low-temperature responsive element. P-box, Gibberellin (GA) responsive element. ABRE, abscisic acid (ABA) responsive element.


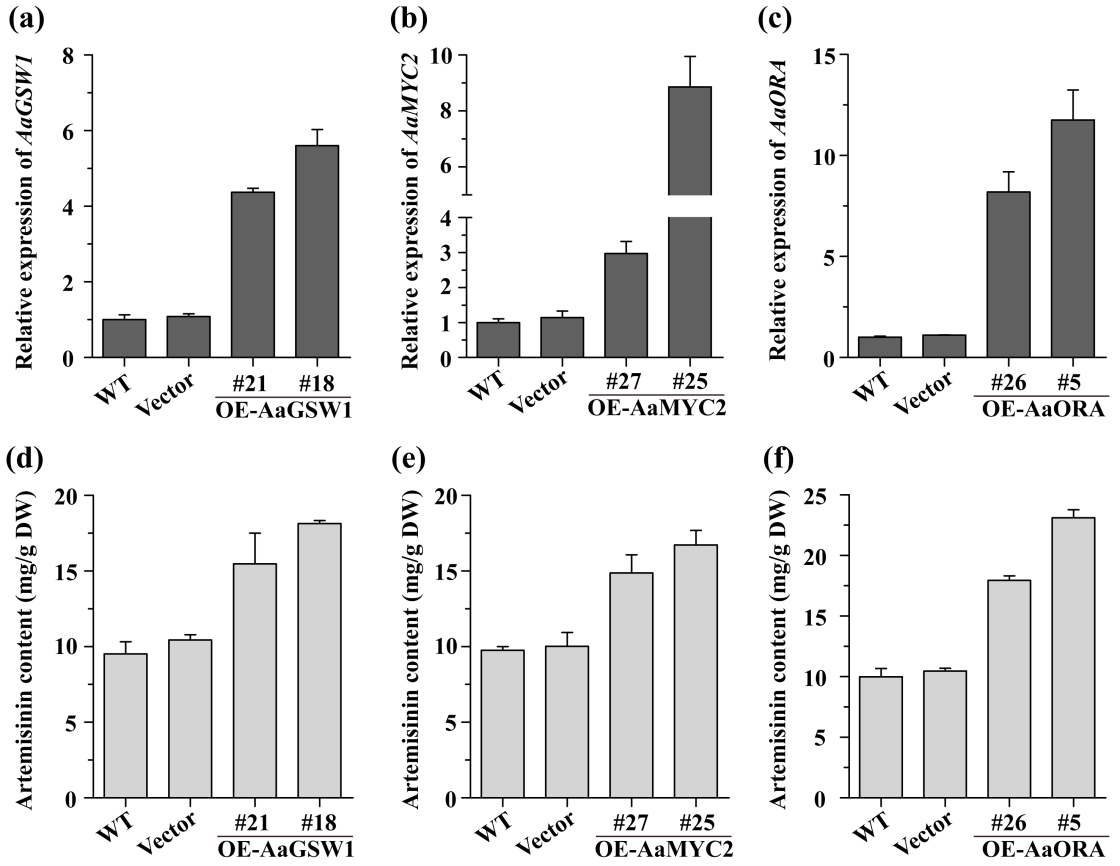


**Figure S7** **Analysis of artemisinin contents in *AaGSW1*, *AaMYC2*, and *AaORA* overexpression *A. annua* plants.**

(a, b, c) Expression of *AaGSW1*, *AaMYC2*, and *AaORA* in the leaves of OE-AaGSW1 (a), OE-AaMYC2 (b), or OE-AaORA (c) transgenic plants, wild-type (WT) plants and plants transformed with the empty vector (control plants, labeled as Vector). *AaActin* was used as an internal reference. Error bars indicate SD (n = 3). (d, e, f) Artemisinin content in the leaves of 3-month-old OE-AaGSW1 (d), OE-AaMYC2 (e), or OE-AaORA (f) transgenic plants, wild-type (WT) plants and Vector control plants was measured by HPLC. The data represent the means ± SD of three replicates from three cutting propagations.

**
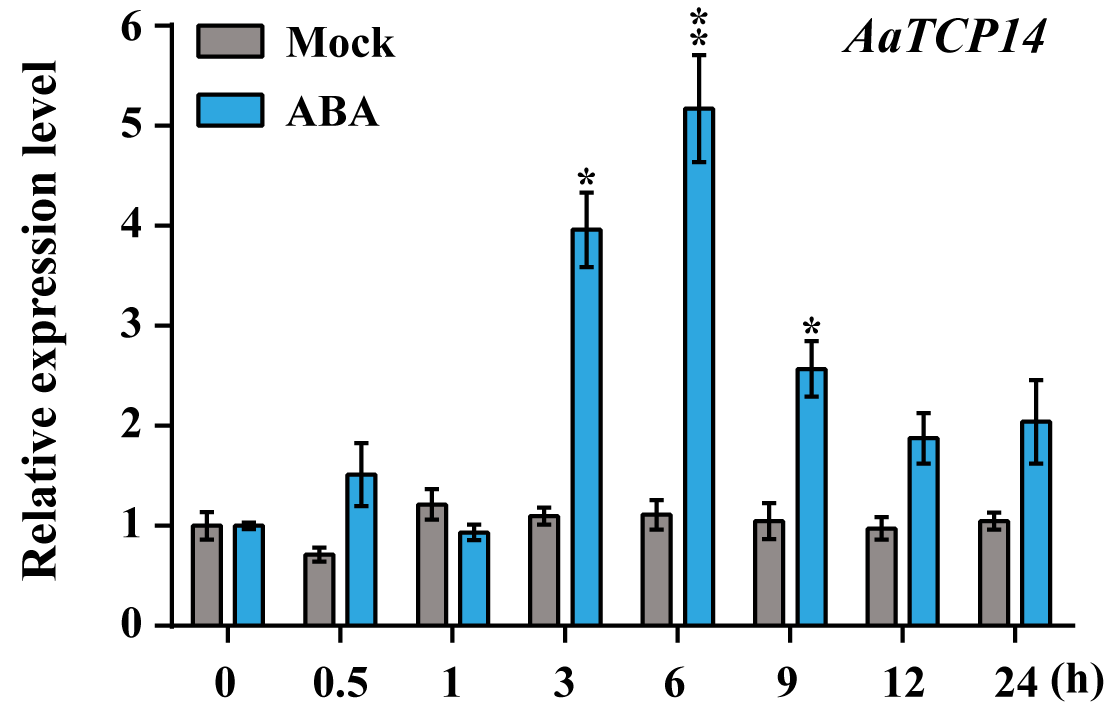
**

**Figure S8 The expression pattern of *AaTCP14* under ABA treatment in *A. annua*.**

Relative expression levels of *AaTCP14* in the leaves of *A. annua* plants treated with mock (0.1% ethanol), and ABA (100 μM) at the indicated times (h). *AaActin* was used as an internal control. The data represent the means ± SD of three replicates from three independent experiments. **P* < 0.05, ***P* < 0.01, Student’s *t*-test.

**Table S1 List of primers used in this study.**

| Primers | Purpose | Primer Sequence (5’-3’) |
| --- | --- | --- |
| 1300-AaTCP15-GFP-F | Plant transformation | GGGGCCCGGGGTCGACATGGATGGTGGTAATGATCA |
| 1300-AaTCP15-GFP-R | Plant transformation | TACCGGATCCACTAGTCGAATTGTGACTTGTACTAT |
| 1300-Anti-AaTCP15-F | Plant transformation | GGGGCCCGGGGTCGACCGAATTGTGACTTGTACTAT |
| 1300-Anti-AaTCP15-R | Plant transformation | TACCGGATCCACTAGTATGGATGGTGGTAATGATCA |
| QPCR-AaTCP15-F | Real-time PCR | TCAATAGGGTTATCTCCGTCAG |
| QPCR-AaTCP15-R | Real-time PCR | TGGTGGTGGTGTTGTTGTG |
| QPCR-antisense-AaTCP15-F | Real-time PCR | CTCCTTCCTTATCCTCCCA |
| QPCR-antisense-AaTCP15-R | Real-time PCR | GGTAGTGGCTTCATCGTCTT |
| QPCR-AaTCP14-F | Real-time PCR | AGGGACGGTATCTACGGGTT |
| QPCR-AaTCP14-R | Real-time PCR | AGCGTGATGCGATTGAGA |
| QPCR-AaTCP11-F | Real-time PCR | TGAGCTGGGACACAAGTCTG |
| QPCR-AaTCP11-R | Real-time PCR | TAGGAGGAATGTCCAGCCGA |
| QPCR-AaTCP16-F | Real-time PCR | AACAAGCCGAACCCTCAGT |
| QPCR-AaTCP16-R | Real-time PCR | AAAGTCCTCTCCTGCGGAA |
| QPCR-AaADS-F | Real-time PCR | GGACTAGGTTCAGGCTATG |
| QPCR-AaADS-R | Real-time PCR | AAGCATGTAATTGACCACC |
| QPCR-AaCYP71AV1-F | Real-time PCR | TCATTTCAGTCGCTT |
| QPCR-AaCYP71AV1-R | Real-time PCR | CCAGTTTGCCTCAGTA |
| QPCR-AaDBR2-F | Real-time PCR | ACTGCTGGTGGCTTTCTTA |
| QPCR-AaDBR2-R | Real-time PCR | ACCCTCGACTTGTTCCTTA |
| QPCR-AaALDH1-F | Real-time PCR | GGACTTGCCTCAGGTGTAT |
| QPCR-AaALDH1-R | Real-time PCR | GTGCCTCTAATCCTTGTTC |
| QPCR-AaMYC2-F | Real-time PCR | TGAGGAAGTTACTGATACAGAATGG |
| QPCR-AaMYC2-R | Real-time PCR | GTATTCCGAATACCTGACCTTGT |
| QPCR-AaGSW1-F | Real-time PCR | TCTCGTCAAAGACACACACATTC |
| QPCR-AaGSW1-R | Real-time PCR | TTGTTCGTAGTTGCTGTAGTGCT |
| QPCR-AaORA-F | Real-time PCR | TCCTCCTCTTCATCCTCTATCG |
| QPCR-AaORA-R | Real-time PCR | CGTTGTCCTTTCTGTCCTCAGT |
| AaActin-F | Real-time PCR | CCAGGCTGTTCAGTCTCTGTAT |
| AaActin-R | Real-time PCR | CGCTCGGTAAGGATCTTCATCA |
| 1391Z-proAaTCP15-F | Promoter Cloned | CCAAGCTTGGCTGCAGCCTTTCATTTTCTTGATTAT |
| 1391Z-proAaTCP15-R | Promoter Cloned | GAATTCCCGGGGATCCGATCACATGAAATAATAATA |
| YFP-AaTCP15-F | Subcellular localization | CGAGCTGCAGGAGCTCATGGATGGTGGTAATGATCA |
| YFP-AaTCP15-R | Subcellular localization | TGCTCACCATACTAGTCGAATTGTGACTTGTACTAT |
| pB42AD-AaTCP15-F | Y1H | TGCCTCTCCCGAATTCATGGATGGTGGTAATGATCA |
| pB42AD-AaTCP15-R | Y1H | TCCAAAGCTTCTCGAGTTACGAATTGTGACTTGTAC |
| pB42AD-AaGSW1-F | Y1H | TGCCTCTCCCGAATTCATGGATAACCAGCTAGATGC |
| pB42AD-AaGSW1-R | Y1H | TCCAAAGCTTCTCGAGCTAGCTCGAAGAATATATTT |
| DBR2pro(3×D):LacZ-F | Y1H | AATTCCACGGTCCCACTTTCACGGTCCCACTTTCACGGTCCCACTTTC |
| DBR2pro(3×D):LacZ-R | Y1H | TCGAGAAAGTGGGACCGTGAAAGTGGGACCGTGAAAGTGGGACCGTGG |
| ALDH1pro(3×A):LacZ-F | Y1H | AATTCTCTGGCCCCACCGTTCTGGCCCCACCGTTCTGGCCCCACCGTC |
| ALDH1pro(3×A):LacZ-R | Y1H | TCGAGACGGTGGGGCCAGAACGGTGGGGCCAGAACGGTGGGGCCAGAG |
| TCP15pro(3×W1):LacZ-F | Y1H | AATTCTGTTCTTGACTACGTTTGTTCTTGACTACGTTTGTTCTTGACTACGTTC |
| TCP15pro(3×W1):LacZ-R | Y1H | TCGAGAACGTAGTCAAGAACAAACGTAGTCAAGAACAAACGTAGTCAAGAACAG |
| TCP15pro(3×W2):LacZ-F | Y1H | AATTCGTAGGTTGACCAATACGTAGGTTGACCAATACGTAGGTTGACCAATACC |
| TCP15pro(3×W2):LacZ-R | Y1H | TCGAGGTATTGGTCAACCTACGTATTGGTCAACCTACGTATTGGTCAACCTACG |
| TCP14pro(3×W3):LacZ-F | Y1H | AATTCTTGCATTGACCACTGATTGCATTGACCACTGATTGCATTGACCACTGAC |
| TCP14pro(3×W3):LacZ-R | Y1H | TCGAGTCAGTGGTCAATGCAATCAGTGGTCAATGCAATCAGTGGTCAATGCAAG |
| pCold-AaTCP15-F | EMSA assay | CGAAGGTAGGCATATGATGGATGGTGGTAATGATCA |
| pCold-AaTCP15-R | EMSA assay | AGATTACCTATCTAGATTACGAATTGTGACTTGTAC |
| Dq Probe-F | EMSA assay | GTCGCTAAACTTGCCACGGTCCCACTTTTTCAACTCTTATTA |
| Dq Probe-R | EMSA assay | TAATAAGAGTTGAAAAAGTGGGACCGTGGCAAGTTTAGCGAC |
| Dq Probe-mutant-F | EMSA assay | GTCGCTAAACTTGCCACGGTAAACATTTTTCAACTCTTATTA |
| Dq Probe-mutant-R | EMSA assay | TAATAAGAGTTGAAAAATGTTTACCGTGGCAAGTTTAGCGAC |
| Aq Probe-F | EMSA assay | GGTTGGTTGCATTATCTGGCCCCACCGTAAACAATCAAATGC |
| Aq Probe-R | EMSA assay | GCATTTGATTGTTTACGGTGGGGCCAGATAATGCAACCAACC |
| Aq Probe-mutant-F | EMSA assay | GGTTGGTTGCATTATCTGGCAAACACGTAAACAATCAAATGC |
| Aq Probe-mutant-R | EMSA assay | GCATTTGATTGTTTACGTGTTTGCCAGATAATGCAACCAACC |
| Topo-AaTCP15-F | BiFC experiment | CACCATGGATGGTGGTAATGATCA |
| Topo-AaTCP15-R | BiFC experiment | CGAATTGTGACTTGTACTAT |
| Topo-AaORA-F | BiFC experiment | CACCATGTTTGCTACTTGCATTCG |
| Topo-AaORA-R | BiFC experiment | AAAAAAAAAAAAGTCATCAT |
| Cluc-AaTCP15-F | Luc complementation | GTCCCGGGGCGGTACCATGGATGGTGGTAATGATCA |
| Cluc-AaTCP15-R | Luc complementation | AGCTCTGCAGGTCGACTTACGAATTGTGACTTGTAC |
| AaORA-Nluc-F | Luc complementation | GGACGAGCTCGGTACCATGTTTGCTACTTGCATTCG |
| AaORA-Nluc-R | Luc complementation | ACGAGATCTGGTCGACAAAAAAAAAAAAGTCATCAT |
| BD-AaTCP15-F | Y2H | GGAGGACCTGCATATGATGGATGGTGGTAATGATCA |
| BD-AaTCP15-R | Y2H | GGATCCCCGGGAATTCTTACGAATTGTGACTTGTAC |
| BD-AaTCP15-△C1-F | Y2H | GGAGGACCTGCATATGATGGATGGTGGTAATGATCA |
| BD-AaTCP15-△C1-R | Y2H | GGATCCCCGGGAATTCTTATTTCTTAGATGGCTCGCCAG |
| BD-AaTCP15-△C2-F | Y2H | GGAGGACCTGCATATGATGGATGGTGGTAATGATCA |
| BD-AaTCP15-△C2-R | Y2H | GGATCCCCGGGAATTCTTAATTGAGTGATGTGAAGTTTG |
| BD-AaTCP15-△N1-F | Y2H | GGAGGACCTGCATATGACATCACTCAATATCTCACT |
| BD-AaTCP15-△N1-R | Y2H | GGATCCCCGGGAATTCTTACGAATTGTGACTTGTAC |
| BD-AaTCP15-△N2-F | Y2H | GGAGGACCTGCATATGCCATCTAAGAAACAACCCCC |
| BD-AaTCP15-△N2-R | Y2H | GGATCCCCGGGAATTCTTACGAATTGTGACTTGTAC |
| BD-AaTCP15-MC-F | Y2H | GGAGGACCTGCATATGCCATCTAAGAAACAACCCCC |
| BD-AaTCP15-MC-R | Y2H | GGATCCCCGGGAATTCTTAATTGAGTGATGTGAAGTTTG |
| BD-AaORA-F | Y2H | GGAGGACCTGCATATGATGTTTGCTACTTGCATTCG |
| BD-AaORA-R | Y2H | GGATCCCCGGGAATTCTCAAAAAAAAAAAAAGTCAT |
| BD-AaORA-△C1-F | Y2H | GGAGGACCTGCATATGATGTTTGCTACTTGCATTCG |
| BD-AaORA-△C1-R | Y2H | GGATCCCCGGGAATTCTCAACGTCTCCTGACTCCTC |
| BD-AaORA-△C2-F | Y2H | GGAGGACCTGCATATGATGTTTGCTACTTGCATTCG |
| BD-AaORA-△C2-R | Y2H | GGATCCCCGGGAATTCTCAACAGCCTATTAAGAGAG |
| BD-AaORA-△N1-F | Y2H | GGAGGACCTGCATATGTTAATAGGCTGTGATGATCG |
| BD-AaORA-△N1-R | Y2H | GGATCCCCGGGAATTCTCAAAAAAAAAAAAAGTCAT |
| BD-AaORA-△N2-F | Y2H | GGAGGACCTGCATATGGTCAGGAGACGTCCGTGGGG |
| BD-AaORA-△N2-R | Y2H | GGATCCCCGGGAATTCTCAAAAAAAAAAAAAGTCAT |
| BD-AaORA-MC-F | Y2H | GGAGGACCTGCATATGGTCAGGAGACGTCCGTGGGG |
| BD-AaORA-MC-R | Y2H | GGATCCCCGGGAATTCTCAACAGCCTATTAAGAGAG |
| AD-AaTCP15-F | Y2H | AGATTACGCTCATATGATGGATGGTGGTAATGATCA |
| AD-AaTCP15-R | Y2H | CACCCGGGTGGAATTCTTACGAATTGTGACTTGTAC |
| AD-AaORA-F | Y2H | TGCCTCTCCCGAATTCATGTTTGCTACTTGCATTCG |
| AD-AaORA-R | Y2H | TCCAAAGCTTCTCGAGTCAAAAAAAAAAAAAGTCAT |
| pGreenII0800-ADS-F | Dual-LUC assay | CGGTATCGATAAGCTTctcgagtatggtgtttcaac |
| pGreenII0800-ADS-R | Dual-LUC assay | ATCCCCCGGGCTGCAGGATTTTCAAAACTTTGAATA |
| pGreenII0800-CYP71AV1-F | Dual-LUC assay | CGGTATCGATAAGCTTaatgggtcaatttcgggttg |
| pGreenII0800-CYP71AV1-R | Dual-LUC assay | ATCCCCCGGGCTGCAGCATGCTTTTAGTATACTC |
| pGreenII0800-DBR2-F | Dual-LUC assay | CGGTATCGATAAGCTTAAGAACTTCGAGATAGAAAA |
| pGreenII0800-DBR2-R | Dual-LUC assay | ATCCCCCGGGCTGCAGTCAGTGATGGAGTTGGTAAA |
| pGreenII0800-ALDH1-F | Dual-LUC assay | CGGTATCGATAAGCTTATGAACCATTAGAAGGGAAG |
| pGreenII0800-ALDH1-R | Dual-LUC assay | ATCCCCCGGGCTGCAGCTTTGTTTTTTATGAAATTT |
| pGreenII0800-PTCP15-F | Dual-LUC assay | CGGTATCGATAAGCTTCCTTTCATTTTCTTGATTAT |
| pGreenII0800-PTCP15-R | Dual-LUC assay | ATCCCCCGGGCTGCAGGATCACATGAAATAATAATA |
| pGreenII0800-PTCP14-F | Dual-LUC assay | CGGTATCGATAAGCTTGCATTGACCACTGAGATGAC |
| pGreenII0800-PTCP14-R | Dual-LUC assay | ATCCCCCGGGCTGCAGGCACGAGTAAACTATCTGCC |
| 1300-AaMYC2-GFP-F | Dual-LUC assay | GGGGCCCGGGGTCGACATGACGATGAATATATGGAA |
| 1300-AaMYC2-GFP-R | Dual-LUC assay | TACCGGATCCACTAGTCCTAGGATCTGACATTCTGT |
| 1300-AaORA-GFP-F | Dual-LUC assay | GGGGCCCGGGGTCGACATGTTTGCTACTTGCATTCG |
| 1300-AaORA-GFP-R | Dual-LUC assay | TACCGGATCCACTAGTAAAAAAAAAAAAGTCATCAT |
| 1300-AaGSW1-GFP-F | Dual-LUC assay | GGGGCCCGGGGTCGACATGGATAACCAGCTAGATGC |
| 1300-AaGSW1-GFP-R | Dual-LUC assay | TACCGGATCCACTAGTGCTCGAAGAATATATTTGCA |
| 1300-AaERF1-GFP-F | Dual-LUC assay | GGGGCCCGGGGTCGACATGATGCAAATGCCTTCGTT |
| 1300-AaERF1-GFP-R | Dual-LUC assay | TACCGGATCCACTAGTACCACTAACGGCTTCACTCA |
| 1300-AabZIP1-GFP-F | Dual-LUC assay | GGGGCCCGGGGTCGACATGAACTACAAGAATTTTGG |
| 1300-AabZIP1-GFP-R | Dual-LUC assay | TACCGGATCCACTAGTCCATGGACCGGAAAGTGTCT |
